# Population genetic insights into establishment, adaptation, and dispersal of the invasive quagga mussel across perialpine lakes

**DOI:** 10.1111/eva.13620

**Published:** 2023-12-08

**Authors:** Linda Haltiner, Piet Spaak, Stuart R. Dennis, Philine G. D. Feulner

**Affiliations:** ^1^ Aquatic Ecology Swiss Federal Institute of Aquatic Science and Technology (Eawag) Dübendorf Switzerland; ^2^ Environmental Systems Sciences ETH Zürich Zürich Switzerland; ^3^ Fish Ecology and Evolution, Center for Ecology, Evolution and Biogeochemistry Swiss Federal Institute of Aquatic Science and Technology (Eawag) Kastanienbaum Switzerland; ^4^ Aquatic Ecology, Institute of Ecology and Evolution University of Bern Bern Switzerland; ^5^ Present address: Department IT services Swiss Federal Institute of Aquatic Science and Technology (Eawag) Dübendorf Switzerland

**Keywords:** ddRADseq, dispersal, Dreissena, phenotypic plasticity, population genetics

## Abstract

Human activities have facilitated the invasion of freshwater ecosystems by various organisms. Especially, invasive bivalves such as the quagga mussels, *Dreissena bugensis*, have the potential to alter ecosystem function as they heavily affect the food web. Quagga mussels occur in high abundance, have a high filtration rate, quickly spread within and between waterbodies via pelagic larvae, and colonize various substrates. They have invaded various waterbodies across the Northern Hemisphere. In Central Europe, they have invaded multiple large and deep perialpine lakes with first recordings in Lake Geneva in 2015 and 2016 in Lake Constance. In the deep perialpine lakes, quagga mussels quickly colonized the littoral zone but are also abundant deeper (>80 m), where they are often thinner and brighter shelled. We analysed 675 quagga mussels using ddRAD sequencing to gain in‐depth insights into the genetic population structure of quagga mussels across Central European lakes and across various sites and depth habitats in Lake Constance. We revealed substantial genetic differentiation amongst quagga mussel populations from three unconnected lakes, and all populations showed high genetic diversity and effective population size. In Lake Constance, we detected no genetic differentiation amongst quagga mussels sampled across different sites and depth habitats. We also did not identify any convincing candidate loci evidential for adaptation along a depth gradient and a transplant experiment showed no indications of local adaptation to living in the deep based on investigating growth and survival. Hence, the shallow‐water and the deep‐water morphotypes seem to be a result of phenotypic plasticity rather than local adaptation to depth. In conclusion, our ddRAD approach revealed insight into the establishment of genetically distinct quagga mussel populations in three perialpine lakes and suggests that phenotypic plasticity and life history traits (broadcast spawner with high fecundity and dispersing pelagic larvae) facilitate the fast spread and colonization of various depth habitats by the quagga mussel.

## 1 INTRODUCTION

Biodiversity provides various benefits to ecosystems, such as enabling ecosystem functioning and acting as buffer against changing, episodic or fluctuating, environmental conditions (reviewed in Chapin et al., 2000; Pires et al., 2018; Srivastava & Vellend, 2005). However, biodiversity itself is threatened by multiple factors such as habitat loss, climate change, pollution, over exploitation, and invasive species (IPBES, 2019). Especially, freshwater systems are most impacted and in danger of loss of biodiversity due to pervasive invasive species (Sala et al., 2000). Furthermore, through transportation of persons or goods, species reach places outside their native range, often facilitated by artificially constructed waterways that connect previously isolated natural populations (e.g., Ponto Caspian macroinvertebrates via Main‐Danube channel; Bij de Vaate et al., 2002). In cases of human‐mediated expansions, invasive species that are most successful show high phenotypic plasticity (reviewed in Davidson et al., 2011), or high adaptive potential to respond to environmental changes (reviewed in Prentis et al., 2008). Understanding the dispersal mechanisms and routes of successful and potentially successful invaders is most relevant for management to prevent further spreads and invasions and the subsequent ecological, economic, and social consequences of invasive species.

One of the ecologically most important invaders for freshwater ecosystems is molluscs, which impact the freshwater ecosystems through controlling primary producers that feed on phytoplankton and seston, graze on periphyton or browse on vascular plants (Strayer, 2010). The globally spreading dreissenids, zebra, and quagga mussels (*Dreissena polymorpha*, Pallas 1771 and *D. bugensis*, Andrusov 1897, also described as *D. rostriformis bugensis*, Wesselingh et al. 2019) are an example of invaders with relevant consequences for the invaded ecosystem. Especially, the quagga mussel affects entire ecosystems due to their large populations, which dominate the heterotrophic biomass (Strayer, 2010). The ability to colonize various substrates and depths, coupled with their fast dispersal between and within lakes, makes quagga mussels successful invaders (Karatayev et al., 2015). Both dreissenids invaded North America in the end of 1980 around the same time, most likely through ballast water (reviewed in Karatayev et al., 2015) and nowadays are found in lakes and rivers from the east to the west coast (Benson et al., 2022). Zebra mussels' native range is wide, including different rivers basins and estuarian reservoirs in the Ponto‐Caspian region (Son, 2007). In Western Europe, zebra mussels started appearing in the early 19th century (Bij de Vaate et al., 2002) and are now found from Finland to Spain (van der Velde & Rajagopal, 2010). Quagga mussels also originating from the Ponto‐Caspian region have started their secondary spread towards Eastern Europe in the 1940's (Orlova et al., 2004; Son, 2007). They were first detected in western Europe in the mid‐2000s (Bij de Vaate et al., 2014). Dreissenids might be able to spread over such large distances, both as adult and as larval stage (Johnson et al., 2001). The widespread invasion of dreissenids has been facilitated by artificial connections of naturally separated watersheds, for example, the Rhine Main Danube canal connecting the Ponto‐Caspian region with Western Europe (Bij de Vaate et al., 2014; Kinzelbach, 1995). Especially, human‐mediated activities like recreational boats and cargo ships (Johnson et al., 2001) transiting this newly built connections allowed dreissenids to spread across long distances. Western European quagga mussel populations likely arrived via the Rhine Main Danube canal from the native region but also from the other invasion front in North America via shipping (Marescaux et al., 2016). In Western Europe, quagga mussels made their way up the Rhine as far as Karlsruhe by around 2006 (Heiler et al., 2013), but were first discovered in Lake Geneva in 2015, in Lake Constance in 2016, and in Lake Neuchâtel in 2017 (Haltiner et al., 2022). Overland transport, for example, via biofouling on recreational boats has been discussed as a potential way of spreading invasive mussels across Switzerland (De Ventura et al., 2016). Survey data highlighted that recreational boats are frequently transport overland between the otherwise unconnected lakes Geneva, Constance, and Neuchâtel, but also from lakes in neighbouring countries (De Ventura et al., 2016).

Like many marine bivalves, dreissenids have a reproduction system, which allows them to spread fast and wide. They are broadcast spawners, fertilization takes place in the water column, prolific breeders with a high fecundity, and have a bento‐pelagic life cycle (Ackerman et al., 1994). This is unusual as fertilization in most freshwater mussels occurs within the female's body and reproduction requires an intermediate host (Haag, 2012). Within a waterbody, quagga mussels' planktonic larvae disperse with currents and streams for up to a month (reviewed in Karatayev & Burlakova, 2022). Once quagga mussels have arrived in a lake, usually they first colonize in the littoral and shallow zone. As the population continues to grow, the mussel spreads deeper into the lake with mussels being recorded as deep as 200 m (Nalepa et al., 2014, 2020). It is still unclear if this is a step‐by‐step or a continuous colonization and if mussels might be pre‐adapted to different depths. Life in the deep requires coping with distinct challenges due to higher hydrostatic pressure, less light, fewer nutrients, and lower but more constant temperature. Indeed, quagga mussels express a different morphology in greater depth: mussels in the deep are more elongated and have a thinner shell and larger siphons (Claxton et al., 1998; Dermott & Munawar, 1993). Analysis based on mitochondrial COI maker, cytochrome *b*, 16s rDNA, RAPD, and microsatellites did not detect genetic differentiation between the two morphological forms suggesting a plastic response rather than a heritable genetic adaptation underlying the morphological differentiation (Claxton et al., 1998; Pavlova et al., 2021; Spidle et al., 1994; Stepien et al., 1999, 2002, 2003). However, we still lack studies that investigate the differences between shallow and deep‐water morphotypes at greater detail utilizing more recent genetic methods with thousands of genome‐wide spread markers.

Microsatellites have been able to detect population structure over large scales (between Western North America and native range populations; Brown & Stepien, 2010) and resolved invasion pathways of quagga mussels on a global scale (Marescaux et al., 2016). However, microsatellites could not detect population structure in quagga mussels between most investigated waterbodies, on a more local scale, such as between Lake Michigan and Lake Huron, USA (Brown & Stepien, 2010). Approaches such as double‐digest RAD (ddRAD) sequencing allow sequencing of the same random regions across the whole genome and recovery of thousands of markers without the need of a reference genome (RADseq: Baird et al., 2008; ddRADseq: Peterson et al., 2012). For the detection of population structure, restiction site‐assosciated DNA sequencing (RADseq) may due to the high number of markers provide a more fine‐scaled resolution in some organisms compared to more traditional approaches based on a limited set of makers (Le Cam et al., 2020; Luikart et al., 2003; Reitzel et al., 2013). Direct comparisons in other organisms have demonstrated that microsatellites might fail to detect genetic structure where approaches like RADseq can pick up signals of genetic differentiation (Ackiss et al., 2020; Sunde et al., 2020). The high number of markers in RADseq approaches may also contain further information about evolutionary processes, for example, that allow the detection of molecular signatures of adaptive evolution (e.g., Morris et al., 2018; Vendrami et al., 2019), which is limited using few microsatellites.

For a highly invasive species such as the quagga mussel, it is important to detect population structure at a high resolution (i.e., using a large number of genome‐wide dispersed markers) on a local scale to reveal mechanisms and routes of local dispersal and identify signatures of putative local adaptation within lakes. Here, we used ddRAD sequencing to investigate population structure between and within lakes in Central Europe. Specifically, we studied recently invaded, unconnected lakes in Switzerland with the aim to explore connectivity for mussel dispersal between lakes. In addition, we utilized a detailed sampling across various depths in Lake Constance to detect outlier loci potentially indicating signatures of adaptive evolution, differentiating morphologically distinct mussels colonizing different lake depths. We further explored local adaptation to depth by performing a reciprocal transplant experiment in the field. In summary, we provide insight into the spread and colonization of quagga mussels among and within large and deep Central European lakes.

## 2 MATERIALS AND METHODS

### 2.1 Mussel sampling

**FIGURE 1:**
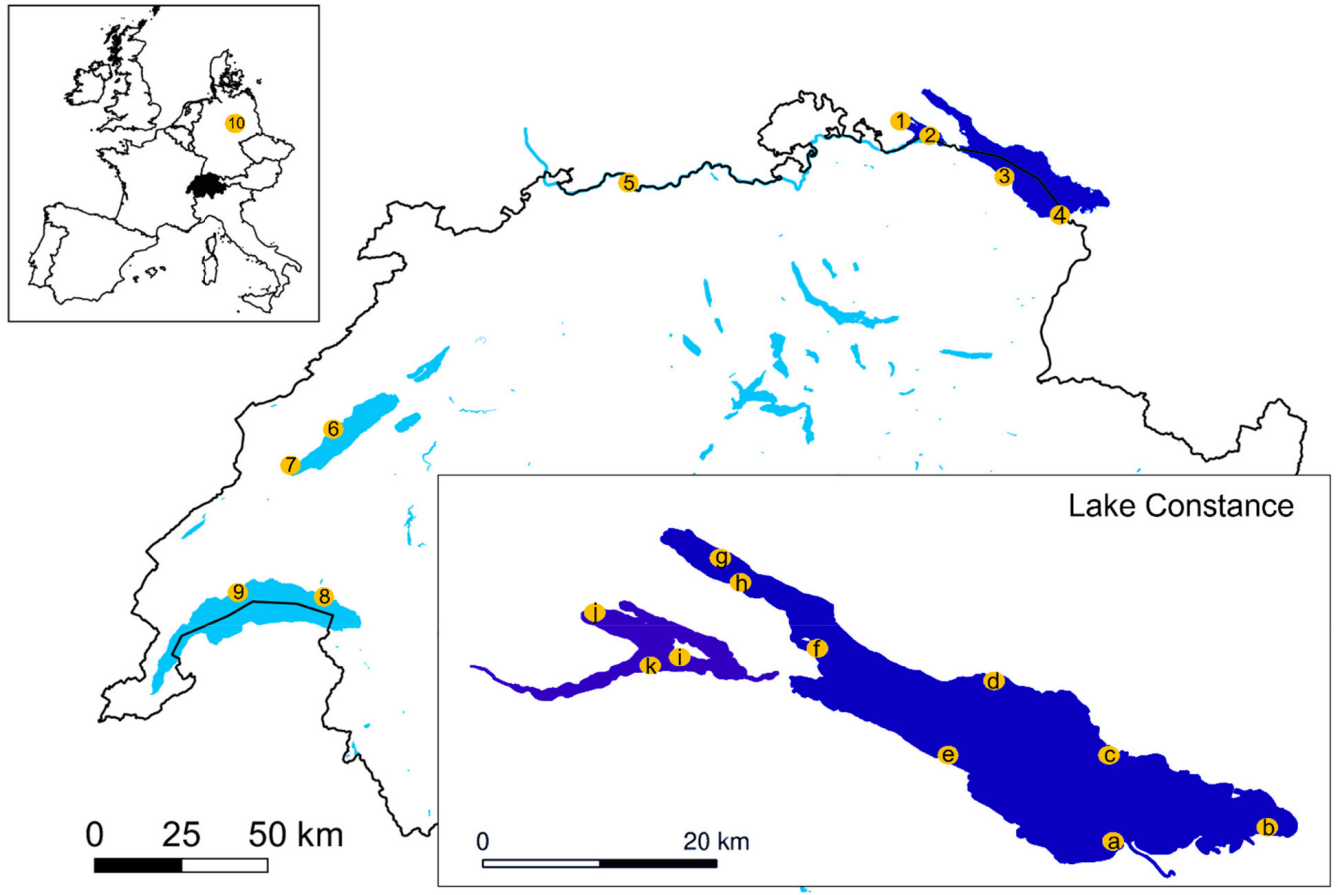
Map of quagga mussel sampling sites across Switzerland including the far German sampling site and outlining the fine‐scale sampling approach in Lake Constance (dark blue). At each site across Switzerland (1–9), mussels were collected by snorkelling. At sites of the fine‐scale sampling in Lake Constance (a–j), two transects were sampled (~500 m apart). Each transect sampled the following depths: 1, 30, 60, and 80 m. In the shallower part in Lower Lake Constance (sites i, j, and k), only 1, 10 m, and max. depth were sampled. Sampling sites: (1) Lower Lake Constance: Radolfzell; (2) Lower Lake Constance: Reichenau; (3) Upper Lake Constance: Uttwil; (4) Upper Lake Constance: Altenrhein; (5) River Rhine: Wallbach; (6) Lake Neuchâtel: St. Aubin; (7) Lake Neuchâtel: Grandson; (8) Lake Geneva: Rivaz; (9) Lake Geneva: St. Prex; (10) Germany: Löebejün. Sites within Lake Constance (a) Altenrhein, (b) Bregenz, (c) Langenargen, (d) Fischbach, (e) Uttwil, (f) Konstanz, (g) Sipplingen, (h) Wallhausen, (i) Reichenau, (j) Radolfzell, (k) Berlingen. Further details about Lake Constance sites in Table S1.

To evaluate dispersal pathways across Swiss lakes, quagga mussels were sampled in June 2019 (Figure 1 and Table 1) on one site in the River Rhine (Wallbach), two sites in Lake Geneva (St. Prex, Rivaz) and Lake Neuchâtel (St. Aubin, Grandson), and on one site in Germany (Löbejün). German samples were collected from a depth of 10 m by a diver. Other locations were sampled by snorkelling in 0.5–2 m depth.

**TABLE 1:** Sequencing information and summary of mussels across Swiss lakes.

Body of water	Site (label in Figure 1)	Coordinates	*N*	No. base pairs	No. polymorphic sites	Nucleotide diversity (*π*)	*N*_e_ effective population size (95% CI) based on pruned no. SNPs
Lower Lake Constance	Radolfzell (1)	47°44′08″ 08°58′02″	13	2,169,649	69,776	0.00992	5066 (5050–5082) 12,386
	Reichenau (2)	47°41′25.5″ 9°03′11.5″	15				
Upper Lake Constance	Uttwil (3)	47°35′07″ 09°20′50″	13	2,171,697	69,684	0.00983	2782 (2770–22,794) 11,247
	Altenrhein (4)	47°29′37.9″ 9°33′1.6″	15				
River Rhine	Wallbach (5)	47°33′52.9″ 7°54′31.0″	14	2,174,076	59,799	0.00988	Infinite 8853
Lake Neuchâtel	St. Aubin (6)	46°54′01″ 6°47′14″	11	2,162,180	52,975	0.00899	Infinite 8540
	Grandson (7)	46°48′43″ 6°39′14″	14				
Lake Geneva	Rivaz (8)	46°28′27″ 6°47′06″	11	2,156,393	54,837	0.00925	239 (236–242) 9384
	St. Prex (9)	46°28′54″ 6°27′41″	8				
Germany	Löbejün (10)	51°37′48″ 11°52′22″	13	2,170,363	52,934	0.00929	472 (infinite CI) 7689

*Note*: *N* = number of individuals per population genotyped and used in population genetic analyses.

In Lake Constance, we increased our sampling effort to also explore the colonization of different sites and depths by the quagga mussel. The lake is separated spatially into Upper, Lower Lake Constance, and Lake Überlingen. Quagga mussels were collected in August 2019 from 11 sites: three in Lower Lake Constance, three in Lake Überlingen, and five in Upper Lake Constance (Figure 1 and Table S1). At each site, we surveyed two transects approximately 500 m apart. The transects included samples in the littoral zone from 1 to 2 m by snorkelling and three replicate Ekman grab samples (grab area 0.0225 m^2^, Hydro‐Bios) at 30, 60, and 80 m depth where possible. At the site Wallhausen, sampling was not possible at 60 m, because a steep drop‐off prevented sampling with the grab sampler, so the sample was collected at 70 m. In Lower Lake Constance, where it is much shallower, grab samples were taken at 10 m and at the maximum depth at each site (20 or 45 m). We sieved the sample through a 2‐mm sieve (Retsch). Live mussels were transported to the laboratory in water in cooling boxes, frozen in liquid nitrogen, and then stored (−80°C). Later, we randomly selected appr. 15 mussels (>8 mm) per site/transect/depth. We also took records of the shell phenotype of these mussels (but we did not use this classification for further analysis): lighter shells were recorded as deep‐water, thicker darker shells as shallow water and intermediated as mixed morphotypes. We dissected the adductor and the foot muscle of the selected mussels and dipped the dissected soft tissue briefly in bleach (NaClO, 1%), ethanol (70%), and autoclaved MiliQ water to remove particles, bacteria, and DNA from the lake water. We froze the dissected soft tissue in Lysis buffer PN (70 μL, LCG Genomics, Berlin).

### 2.2 DNA extraction

DNA of 731 dreissenid individuals was extracted following the LGC‐sbeadex tissue protocol (LGC Genomics) with minor adjustments: for lysis, we used the PN buffer instead of the standard lysis buffer and we degraded proteins with Proteinase K (15 μL, 20 mg/mL) during 3 h at 55°C on a thermo shaker (BioShake iQ) with 1000 rpm rotation instead of the standard Protease overnight. After lysis, we added an RNA digestion step by adding 4 μL RNase (10 ng/μL) for 30 min at 37°C before we followed the bead‐based DNA extraction protocol of LCG using the extraction robot King Fisher (Thermo Scientific). DNA concentration of each sample was measured using a Spark 10 m Multimode Microplate Reader (Tecan). Each sample was standardized to a DNA concentration of 7.5 ng/μL using a liquid handling system.

### 2.3 Library preparation and sequencing

We prepared 17 ddRAD libraries following the protocol by Peterson et al. (2012) with minor adjustments. We distributed 727 experimental individuals randomly across the 17 libraries (each containing 42–48 individuals). Each library further contained a sample of the same individual (UN‐3187) to allow to quantify genotyping error while sequencing and a negative control (pure water). 200 ng DNA per individual was digested with the two restriction enzymes EcoRI and TaqI. For 45 individuals with lower DNA content, enzymatic digested was started with 65–100 ng DNA. After we ligated the barcode adapters to each individuals' sample, we combined individuals into 17 libraries (42–48 individuals per library). We selected our target length fragments (550 bp long) with AMPURE XP beads (Beckman Coulter, 0.6× and 0.12×) and continued only with fragments with biotinylated P2 adapters. PCR was performed using the KAPA HiFi HotStart ReadyMix (Roche) to amplify DNA and add 17 different Illumina primers combination that identified the 17 libraries. We then purified the libraries and measured fragment lengths (mean fragment length = 561 bp) using a 2200 TapeStation (Agilent) and combined them into two final pools. The libraries were sequenced on three lanes and flow cells on an Illumina NovaSeq 600 with 2 × 150 bp paired‐end sequencing and produced a total of 1.8 billion raw reads.

### 2.4 Demultiplex and mapping

We followed the dDocent pipeline (Puritz et al., 2014) for demultiplexing and mapping. Reads were demultiplexed with the stacks software 2.41 using *process_radtags* function. We removed 11 samples with fewer reads (1430–144,228 reads) than the negative controls (average number of reads for negative controls *n* = 17 was 10,406, ranging from 1136 to 151,134 reads). Then, all the reads were mapped against the quagga mussel reference genome (GCA_007657795; Calcino et al., 2019) using bwa 0.7.17 and quality filtered afterwards using sambamba 0.7.1. Eight individuals with less than 96% mapped reads were discarded as well as 47 zebra mussels (70%–75% mapped reads; note that these zebra mussel individuals were accidentally added and not further used in the subsequent analyses). This resulted in a data set of mapped reads for 680 quagga mussels covering all sampling locations.

### 2.5 SNP calling, filtering, and genotyping error

For SNP calling and downstream analysis, we created two datasets to address our two distinct research questions. To evaluate the genetic structure of quagga mussels across Swiss lakes, we used 87 individuals originating from four sampling locations: Lake Geneva, Lake Neuchâtel, Rhine, Germany (see Figure 1 for sampling map and Table S2 for individual information), as well as 56 individuals from the larger sampling in Lake Constance. We treated Lower and Upper Lake Constance as separate lakes basins, although they are connected (Upper Lake Constance flows into Lower Lake Constance). We selected individuals collected at 1 m for two sites in each basin. Hereafter, we will refer to this data set as “across Swiss lakes.” For the analysis within Lake Constance, we used all 581 individuals originating from 11 different sampling sites and different depths (1, 10, 30, 60, 70, and 80 m, see Figure 1 for sampling map and Table S3 for individual information). Hereafter, we will refer to this data set as “within Lake Constance.”

SNPs were called from mapped reads separately for each data set using freebayes v 1.3.1 (Garrison & Marth, 2012). We called 10,576,951 variants for the across Swiss lakes and 16,614,375 variants for the within Lake Constance data set. Variants were filtered following the dDocent filtering tutorial (Puritz et al., 2014) using the software VCFtools (Danecek et al., 2011) and vcflib except for the Hardy–Weinberg and rad‐haplotyper filter. Default parameter settings were applied, with the following exceptions: the individuals missing genotypes (across Swiss lakes analysis: *‐‐remove lowDP > 0.7*; within Lake Constance analysis: *‐‐remove lowDP > 0.85*), the genotype missingness (across Swiss lakes analysis: *‐‐max‐missing 0.95*; within Lake Constance analysis: *‐‐max‐missing 0.93*), minor allele frequency (*‐‐maf 0.01*), minimum mean depth of genotypes (*‐‐min‐meanDP 10*), maximum mean depth (across Swiss lakes: *‐‐max‐meanDP 50*; within Lake Constance analysis: *‐‐max‐meanDP 40*), keeping only biallelic sites (*‐‐min‐alleles 2 ‐‐max‐alleles 2*). After filtering, we removed indels and the final datasets contained 81,197 variants (across Swiss lakes: 127 individuals), and 4939 (within Lake Constance: 549 individuals) (see Table S4 for detailed filtering steps).

The inclusion of the same individual in every library allowed the assessment of genotyping error for each of the two datasets. We used the program Tiger to calculate genotyping error in the individual sequenced in all 17 libraries (Bresadola et al., 2020).

To verify whether results might be influenced by potential linkage between SNPs, we produced datasets thinning the number of linked SNPs to one SNP per 150 bp (RADtag length using VCFtools *‐‐thin 150*). Using these thinned datasets, we repeated analyses that could be affected by non‐independence of SNPs (pairwise *F*_st_ estimates, PCA, and LEA clustering).

### 2.6 Population structure and differentiation across Swiss lakes

We explored genetic diversity for each of the six populations (Lower Lake Constance, Upper Lake Constance, Rhine, Lake Neuchâtel, Lake Geneva, Germany) by calculating nucleotide diversity (*π*) across all variable sites in VCFtools and dividing it by the overall number of sequenced sites. Population structure across populations was assessed via principal component analysis (PCA) with the R package SNPRelate (Zheng et al., 2012) and via admixture analysis using the R Package LEA (Frichot & François, 2015; R version 4.1.1; R Core Team, 2017). The admixture analysis was run using the snmf function in LEA for *K* = 1–10. For each value of *K*, we run 10 independent runs. Based on the entropy criterion, we chose *K* to assess the number of ancestral populations that best explained the genotypic data (Frichot et al., 2014). Results were visualized using R, and individuals were ordered by population. Effective population size (*N*_e_) was estimated for each population basin pruned for linkage disequilibrium (in plink using *‐‐indep 50 5 1.01*) with the linkage disequilibrium method in NeEstimator v.2.1 and *p*_Crit_ values of 0.05 (Do et al., 2014). To estimate population differentiation, we calculated pairwise *F*_st_ values between populations using Arlequin v3.5.2.2 (Excoffier & Lischer, 2010) and tested if values significantly differed from zero via the implemented permutation test (>9999 permutations). To convert vcf files to Arlequin input format, we used PGDSpider v2.1.1.5 (Lischer & Excoffier, 2012).

### 2.7 Population structure, differentiation, and detection of loci associated with depth within Lake Constance

Population structure within Lake Constance was assessed in the same way as in the other data set using PCA and admixture analysis across sites and depths within Lake Constance. To estimate the population differentiation within Lake Constance, we calculated pairwise *F*_st_ values between sampling sites and categorized sampling depths into three depth categories: 1 m (including depths of 1–10 m, *N* = 308), 30 m (including depths of 20–45 m, *N* = 151), and 60 m (including 60–80 m, *N* = 90). The same three depth categories were also used to identify outlier loci indicative of local adaptation. While we recorded phenotypic information (see phenotype column in Tables S2 and S3), we did not make use of this more subjective category in any of our analyses, but rather based our analyses on the collection depth of the individuals.

We performed two analyses to detect possible loci suggestive of local adaptation to depth. We used an *F*_st_ outlier approach utilizing the R package outFlank (Whitlock & Lotterhos, 2015) and an ecological association study as implemented in LEA (Frichot & François, 2015). Sampling depths were categorized in both analyses (see details above for the three depth categories and respective sample sizes). Outflank identifies putative candidate loci under selection to depth with a maximum likelihood approach that generates a *F*_st_ distribution of neutral loci by trimming extreme *F*_st_ values. To generate this distribution, we followed the authors' guidelines and used a subset of quasi‐independent SNPs (LD pruned in plink using *‐‐indep 50 5 1.01*). A latent factor mixed models in LEA were used to search for associations between individual SNPs and sampling depth as an environmental variable. We ran the model with the latent factor *K* = 3 (iterations = 10,000, burning: 5000). We combined *Z* scores of five repetitions and adjusted *p*‐values for multiple testing using a Benjamini–Hochberg procedure and an expected false discovery rate of *q* = 0.05.

Allele frequency of all potential candidate loci was calculated across depth using vcftools (*‐‐freq*). We also calculated allele frequency across sampling sites at these same loci to estimate the amount of drift on these loci. Candidate loci on the same RADtag (sequenced fragment of 150 bp) were treated as one and only candidate loci that showed a gradual allele frequency change along depth were considered further.

For loci identified with both approaches and the loci with the highest allele frequency changes between depths from the latent factor mixed model, we performed a blast search to investigate whether these candidate loci were close to gene coding regions. For these loci, we blasted their sequence of the reference genome between a pair of RADtags (a length between 270 and 350 bp, NCBI database; Altschul et al., 1997). Only sequences that matched the quagga mussel sequence by 80% or more were reported.

### 2.8 Reciprocal transplant experiment: Study area and sampling

We performed two reciprocal transplant experiments transplanting mussels within a lake between different depths, one in Lake Geneva and one in Lake Constance (LG: N: 46°30′0.82″, E: 6°39′39.01″, depth: ca. 110 m, LC: N: 47°45′43.68″, E: 9°7′50.02″, depth: ca. 140 m). Mussels for the experiment were collected from a shallow and a deep environment in both lakes: 10 m (±3 m) and 60 m (±6 m). We used a bottom trawl specifically made for collecting mussels from soft sediment (mesh size: 7 mm, dredging distance: appr. 100 m). In Lake Geneva, we collected mussels in front of Lausanne (10 m: N: 46°30′34″, E: 6°36′41″ and for 60 m: N: 46°30′19″, E: 6°36′23″), and in Lake Constance, we sampled between Konstanz Egg and the island of Mainau (10 m: N: 47°41′37″, E: 9°11′59″ and 60 m: N: 47°41′51″, E: 9°12′24″). Mussels retrieved from the dredge were stored in water and sorted into two size classes, 8–12 and 12–16 mm. We used different size classes to reduce inferring effects of size.

### 2.9 Reciprocal transplant experiment: Experimental set‐up

Quagga mussels of both origins (10 and 60 m) were reciprocally transplanted between the two experimental depths (10 and 60 m) within a lake. So, the experimental design had two destination treatments and two source treatments (in total four transplant treatments: (1) mussels from shallow origin to a shallow destination, (2) mussels from shallow to deep, (3) mussels from deep to shallow, and (4) mussels from deep to deep). Approximately 150 adult mussels were used for each treatment: 15 bags (fibreglass, mesh size: 1.4 mm) of five individuals per size class. Each treatment was split up on three ropes (five bags per rope). Bags were attached to ropes in the open water. On day one of the experiment, we took pictures of all mussels to assess their length. As an indication for local adaptation, we assess survival and shell length in the new environment after 17, 35, 75, and 100 (±5 days) days. On assessment days, mussels were taken out of the bags, placed in plastic trays, and left undisturbed for approximately 10 min in lake water. During this time, mussels could recover from handling stress. After 10 min, survival was assessed. Mussels were classed as alive when siphon or foot was visible, or mussel shell closed when touched. Dead mussels were sorted out and stored in ethanol. We took photos of the living mussels to assess shell size.

### 2.10 Reciprocal transplant experiment: Statistical analysis

As survival was overall low in 10 m due to manual or non‐natural events like shearing against the rope or while handling, we focused our survival analysis in 60 m depth. We fitted an accelerated failure time survival model (AFT, Weibull regressions) to mussel survival data to obtain predicted survival times for different treatments. We used lake and mussel's depth of origin (10 or 60 m) as predictors. We used the R package survival to fit the AFT model (Therneau, 2015). We further analysed if mussel growth from both origins differs if reared in both destination habitats during 98 days in Lake Constance and 104 days in Lake Geneva. We used a GLM in the R package glmmTBM (Brooks et al., 2017) using growth (individual size at end – mean size per bag at start) as response variable and assessed its dependence on depth of origin, depth of destination, lake, the interaction of lake and destination, and bag nested in rope as random variables to exclude variation across bags and ropes using a normal distribution. Model assumptions were met and checked with the package DHARMa (Hartig, 2022). We also investigated for competition‐induced density‐dependent growth. We did not see changes in mean or variance in sizes at different mussel densities indicating no signs for competition in our data (Figure S1); hence, we did not include density of survivors in the model.

## 3 RESULTS

In the across Swiss lakes data set, we successfully genotyped 127 individual quagga mussels. The within Lake Constance data set contained 549 individuals. The addition of the same individual in each of our 17 libraries allowed a thorough assessment of genotyping error for both data sets. The mean error rate (weighted for read length) was calculated across libraries and resulted in small error rates that is, 0.006% in the across Swiss lakes data set and 0.005% in the within Lake Constance data set, suggesting that our genotype filtering strategy was successful and resulted in highly reliable genotypes.

### 3.1 Genetic diversity across Swiss lakes

Across the 127 analysed quagga mussels, we detected 81,197 SNPs (variable sites with minor allele frequency >1%) and SNPs on average had a read depth of 14.6×. Missing data per individual was on average 3% (ranging from 0% to 16%). We evaluated genetic diversity for each population (here each lake or river) and compared between the six populations (lakes Geneva, Neuchâtel, Germany, River Rhine, and Lake Constance – split into Lower and Upper Lake Constance) by using the sequencing information available (range number of SNP per population: 52,934–69,776; see Table 1).

Average nucleotide diversity (*π*) for the six populations ranged between 0.00899 (Lake Neuchâtel population) and 0.00992 (Lower Lake Constance population). Hence, our analysis revealed only minor variation in genetic diversity between populations (see Table 1).

Effective population size (*N*_e_) ranged from 239 in Lake Geneva to 5066 in Upper Lake Constance and reached infinite values in Lake Neuchâtel and River Rhine (see Table 1). Infinite values indicate that sampling error explains the results and thus suggests that too few individuals were sampled to estimate *N*_e_ (Do et al., 2014).

### 3.2 Population structure and differentiation across Swiss lakes

**FIGURE 2:**
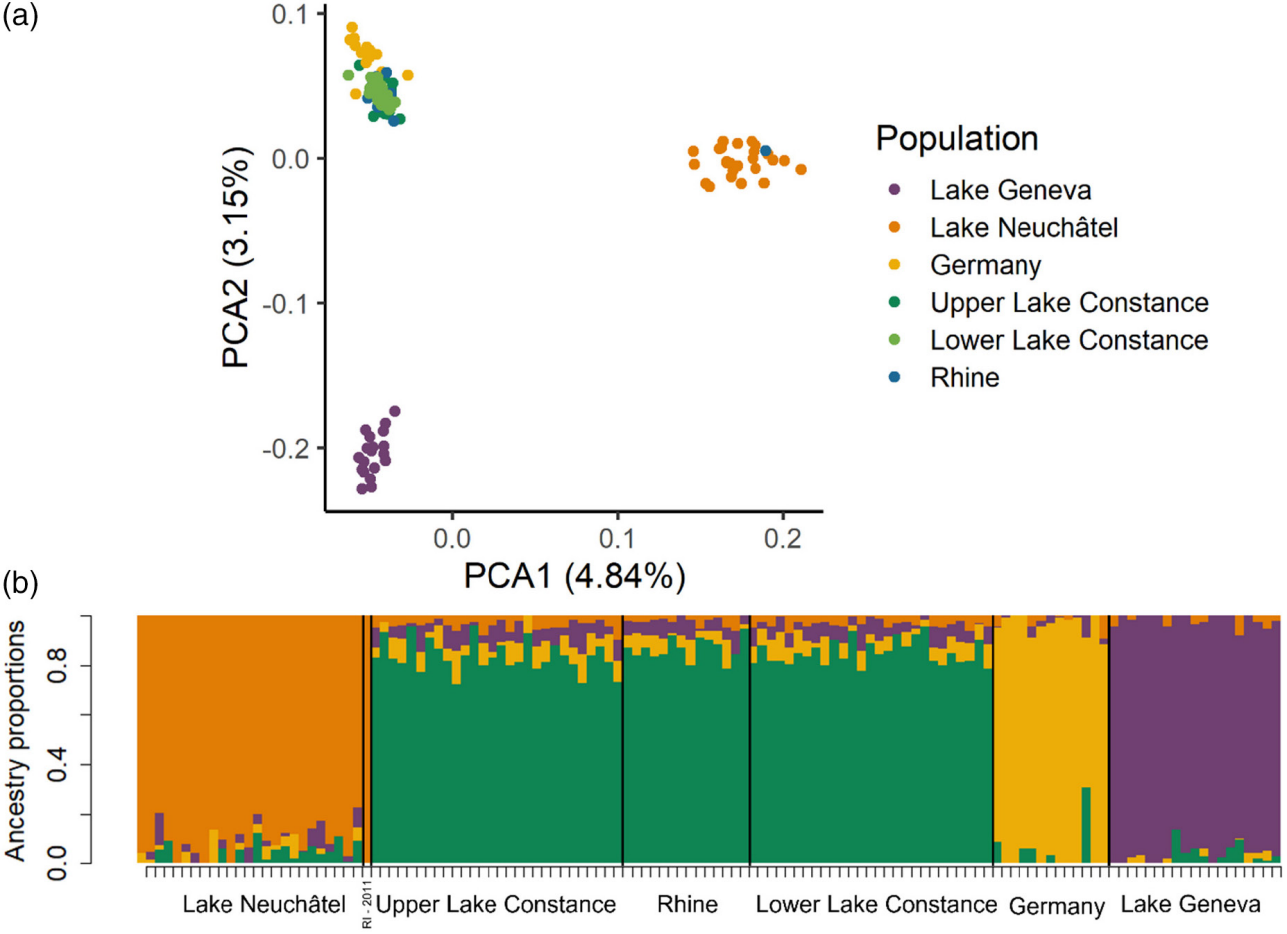
Genetic differentiation between quagga mussels from Lake Geneva, Lake Neuchâtel, and the Constance regions (Upper and Lower Lake Constance, Rhine, and Germany). (a) PCA plot showing the genetic differences between 127 quagga mussels based on 81,197 loci. Populations are coloured differently (see legend). Quagga mussels from Upper and Lower Lake Constance are not distinct from River Rhine mussels, while Lake Geneva and Neuchâtel populations form separate groups. (b) Quagga mussels' genetic composition (admixture proportions estimated by LEA). Lake Constance and River Rhine populations show similar ancestry proportions. Each bar represents an individual (ordered by lakes), and the colours represent the proportion of each of the four genetic contributions.

A PCA based on the 127 genotypes resolved three none‐overlapping clusters separating the mussels from Lake Neuchâtel, Lake Geneva, and a cluster comprised of individuals from Lower and Upper Lake Constance, Rhine, and Germany (Figure 2a). While not fully distinct, mussels from Germany are differentiated from Lake Constance mussels along the second axis explaining 3.16% of variation. The first axis explains 4.75% of the variation and separates Lake Neuchâtel from all other populations. Furthermore, principal components (3–11) have been inspected and do not provide any additional resolution or insights.

The admixture analysis identified four genetically distinct cluster with the cross‐entropy criterium being lowest at *K* = 4 (Figure S2). The admixture plot allows to distinguish mussels from Lake Neuchâtel, Lake Geneva, and Germany but clusters mussels from Lower and Upper Lake Constance and the Rhine together (see Figure 2b). When the data were segregated into five clusters (*K* = 5), samples from Lake Neuchâtel get split into two clusters but the different sampling sites within Lake Constance and the Rhine were not separated (Figure S3).

Both the PCA and admixture analysis identified one individual (RI‐2011) sampled in the Rhine River but genetically clustering with all the individuals sampled in Lake Neuchâtel. We restrain from speculating about a biological explanation due to this single occurrence without any evidence of admixture, which might be explained by a laboratory mistake on our side.

The data set with one SNP per 150 bp RADtag comprised 14,300 SNPs. The results of PCA and admixture analysis were overall consistent with those from the full dataset (Figure S4).

Genetic differentiation between populations was absent between Upper Lake Constance, Lower Lake Constance, and the Rhine (all pairwise *F*_st_ ≤ 0). All other populations were significantly genetic differentiated from each other (*p* << 0.001). The highest differentiation was observed between mussels from Lake Neuchâtel and Lake Geneva. Differentiation between Germany and Upper Lake Constance, Lower Lake Constance, and the Rhine was low but statically different from zero (see Table 2). Estimates using the dataset with one SNP per 150 bp resulted in qualitatively the same results (minor changes in pairwise *F*_st_ values, same results in significance testing).

**TABLE 2:** High genetic differentiation (pairwise *F*_st_ values) across mussels from Swiss lakes.

Pairwise *F*_st_ values	Lake Neuchâtel	Lake Geneva	Germany	River Rhine	Upper Lake Constance	Lower Lake Constance
Lake Neuchâtel	–	0.10	0.10	0.05	0.06	0.06
Lake Geneva	0.00	–	0.08	0.08	0.04	0.05
Germany	0.00	0.00	–	0.04	0.04	0.04
River Rhine	0.00	0.00	0.00	–	0.00	0.00
Upper Lake Constance	0.00	0.00	0.00	0.04	–	0.00
Lower Lake Constance	0.00	0.00	0.00	0.40	0.13	–

*Note*: Above diagonal *F*_st_ values are given, and below *p*‐values indicate which values are significantly different from zero.

### 3.3 Population structure and differentiation within Lake Constance

The within Lake Constance data set contained 549 individuals from 11 sites across multiple depths. Our filtering approach resulted in 4939 SNPs with an average depth of 13.1×. As the data set contained many individuals but a relatively low coverage, our missing data criterion largely reduced the number of SNPs (Table S4). Missing data per individual was on average 5% (ranging from 0% to 58%).

**FIGURE 3:**
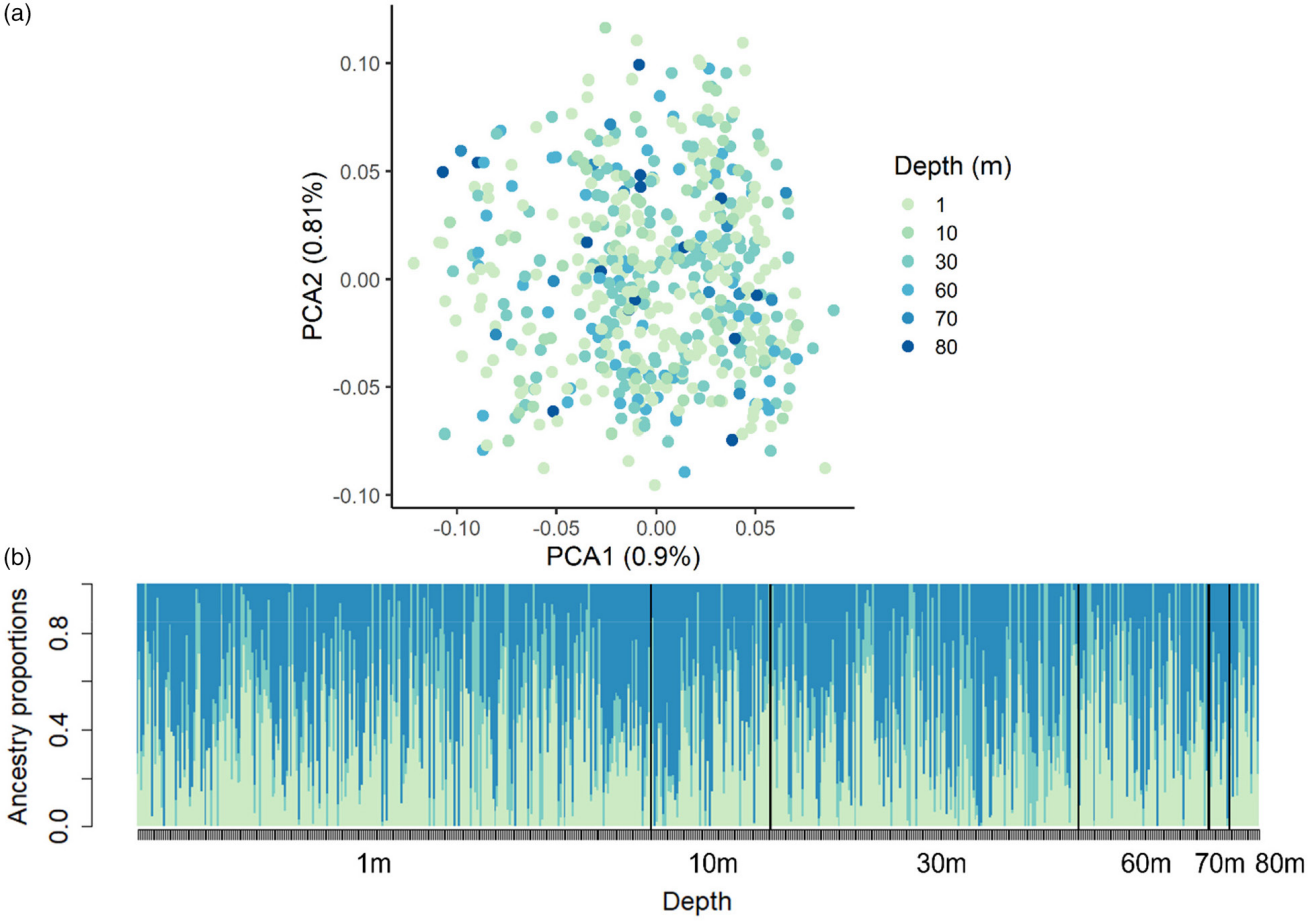
No genetic differentiation between quagga mussels sampled across depth gradients in Lake Constance. (a) PCA plot showing the genetic similarities between 549 quagga mussels based on 4939 loci. Colours indicate different collection depths. (b) Quagga mussels have similar admixture over all depths (admixture proportions estimated by LEA). Each bar (ordered by sampling depth) represents an individual, and the three colours represent the proportion of each of the three genetic contributions.

The PCA for mussels based on 549 genotypes at 4939 loci did not split the two lake basins, nor sites within the lake, nor individuals by depth (see Figure 3). The first two principal components explained 0.9% and 0.81% of the total variation across 549 individuals.

For the admixture analysis, the cross‐entropy criterion showed a minimum at *K* = 9 (Figure S5). We show *K* = 3 sorting individuals by the categorized sampling depths of 1, 30, and 60 m (Figure 3). Neither *K* = 3 (equal to categorized sampling depths) nor *K* = 9 (closer to the 11 sampling sites) could distinguish biologically meaningful populations (Figure S6 for *K* = 9).

The dataset with one SNP per 150 bp RADtag comprised 796 SNPs. The results of PCA and admixture analysis were overall consistent with those from the full data set (Figure S7).

There was no genetic differentiation between sampling sites in Lake Constance (all pairwise *F*_st_ ≤ 0). There was also no genetic differentiation between the three depth categories (all pairwise *F*_st_ ≤ 0). We also detected no genetic differentiation between depth categories at individual sites (all pairwise *F*_st_ ≤ 0). Estimates using the dataset with one SNP per 150 bp resulted in qualitatively the same results (minor changes in pairwise *F*_st_ values, same results in significance testing).

### 3.4 Genomic signatures of local adaptation in Lake Constance

**FIGURE 4:**
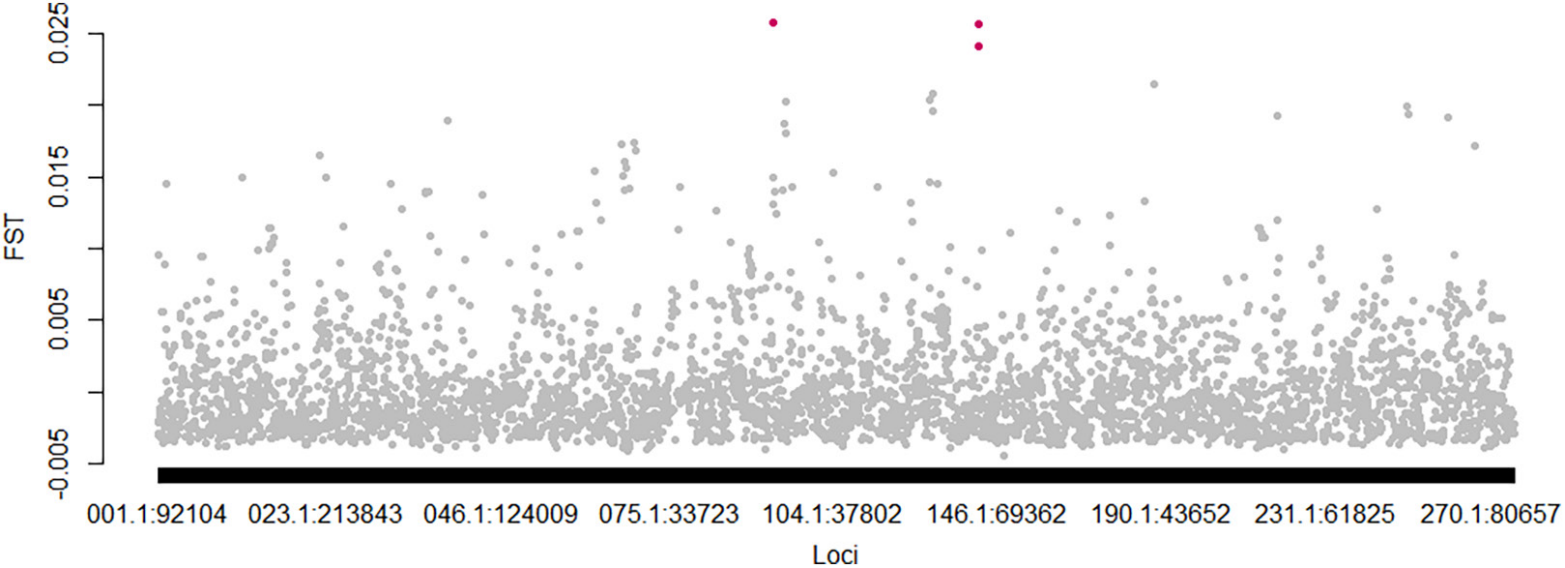
Genome scan detecting three candidate loci detected potentially shaped by selection along a depth gradient. *F*_st_ values of loci along the chromosome as estimated by outflank. Detected candidate loci are marked in pink and represent the highest *F*_st_ values.

We used two different approaches (outFlank and LEA) with the aim to identify candidate loci consistent with a putatively non‐neutral behaviour, with an exceptionally high level of spatial differentiation along a depth gradient. The first approach, outFlank, identified three candidate loci showing exceptionally high differentiation (Figure 4). Two of which are located less than 150 bp apart which we treated as one candidate locus, since they are inherited together and were targeted by the same RAD fragment during sequencing. We calculated allele frequency across sampling depths (see Table S5). The third candidate locus did not gradually change its allele frequency along depth and was therefore not further considered.

In the latent factor mixed model from LEA, we chose a *K* = 3 reflecting the three different depth categories. This was justified as the cross‐entropy criterion showed the strongest decrease between *K* = 1–3. After correcting for false discovery rate (*q* = 0.05) and an inflation factor (lambda = 0.43), 59 SNPs were identified as candidate loci putatively associated with depth. Allele frequency changes between depths ranged between 7.2% and 14.9%. Six loci out of the 59 had no gradual allele frequency change between depths and were not further considered.

To set the allele frequency changes between depths into perspective, we estimated frequency changes between sites and revealed that those potential candidate loci differed more in frequency between sites (range between 8.4% and 30.6%) than between different depths.

Only one locus was identified in both approaches, LEA and outFlank (VMBQ0100136.1389253) with an allele frequency change between depths of 14.5%. The locus with highest allele frequency changes of 14.9% between depths in the latent mixed model was locus VMBQ0100256.1193498. However, maximal allele frequency changes between the 11 sampling sites at these two loci was 15.6% (between sites Radolfzell and Konstanz) and 23.2% (between sites Reichenau and Altenrhein) showing more changes across sites than across depths. For these two candidate loci, a BLAST search was performed but utilizing the whole RADtag sequences (270–340 bp) of these two loci did not identify any sequence similarity with any functional gene region.

### 3.5 Evaluating local adaptation with a reciprocal transplant experiment

**FIGURE 5:**
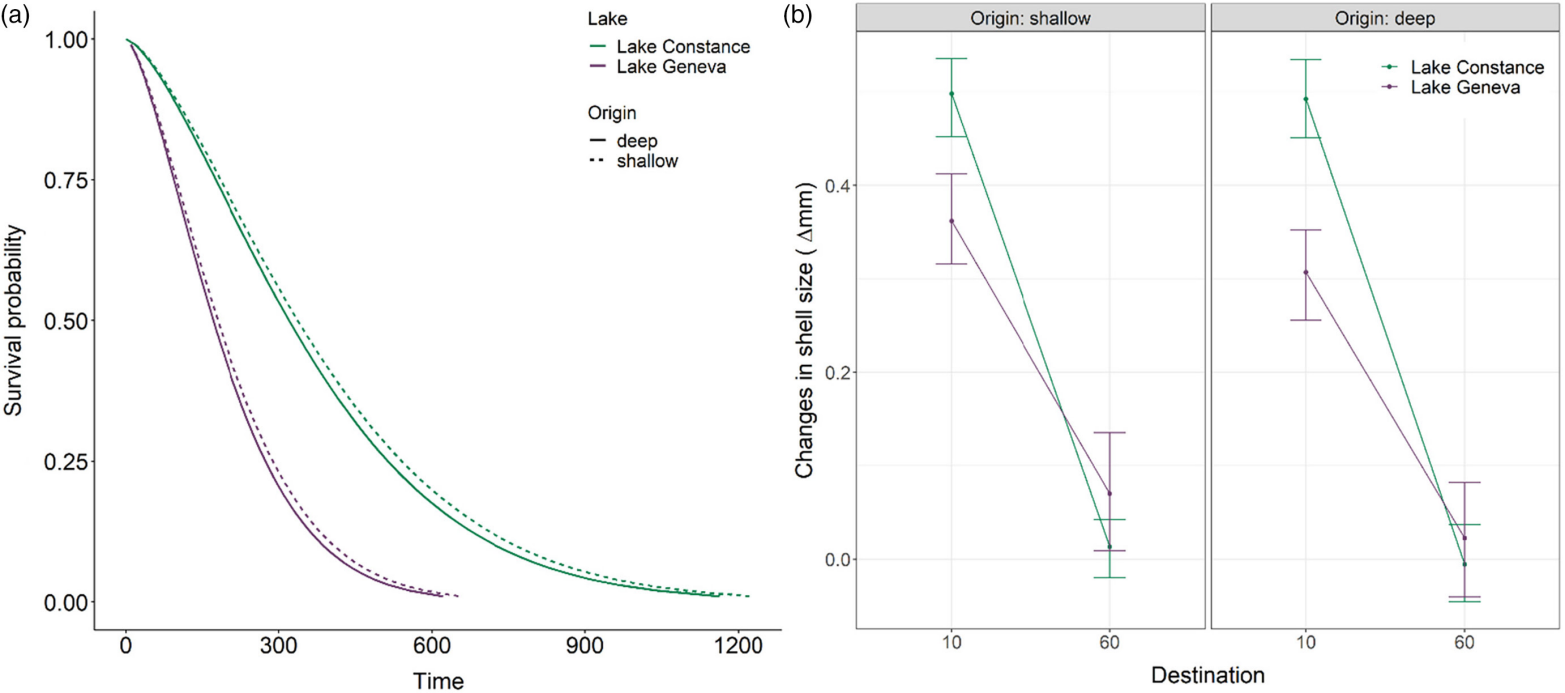
Reciprocal transplant experiment revealed no evidence for local adaptation at different depth. (a) Predicted survival probability of quagga mussels in the pelagic in 60 m depth in Lake Constance (green) and Lake Geneva (purple). Dashed lines show the survival of transplanted quagga mussels from a shallow environment (10 m), and the solid line shows the survival of mussels collected in 60 m in the depth of destination of 60 m. All lines show fitted values of AFT survival models (Weibull regressions). (b) Mussel size changes as shell size differences (in mm, mean ± 95% confidence interval) between start and end of the reciprocal transplant experiment in Lake Constance (for 98 days in green) and in Lake Geneva (for 104 days in purple).

To support the genome scan for local adaptation, we evaluate the reciprocal transplant experiment conducted in Lake Constance and Lake Geneva. In the survival analysis, we found that in 60 m less mussels survived in Lake Geneva then in Lake Constance irrespective of their depth of origin. In Lake Constance, 50% predicted survival probability for deep‐water mussels was almost as long (337 days) as for shallow‐water mussels (320 days). In Lake Geneva, mussels reached the predicted 50% mortality 150–185 days earlier than in Lake Constance (see Figure 5a).

While analysing growth of mussels from different depths reared in 60 m, we found that mussels grew faster in the shallow environment (raw data mean growth = 0.43 mm, SD = 0.25 mm) than in the deep (0.02 mm, SD = 0.25 mm, Figure 5b, for mussel sizes over time see Figure S8). Growth was not affected by the depth of origin (*z* = −1.69, *p* = 0.09). Overall, mussels grew more in Lake Constance (0.25 mm in 98 days, SD = 0.33 mm) than in Lake Geneva (0.19 mm in 104 days, SD = 0.30 mm, *z* = −6.7, *p* < 0.005). The interaction between lake and destination was statistically significant (*z* = 6.0, *p* < 0.005).

## 4 DISCUSSION

### 4.1 High genetic differentiation between and similar genetic diversity across Swiss lakes

Across Swiss lakes, our high‐resolution ddRAD data revealed strong genetic differentiation between quagga mussels from Lake Neuchâtel, Lake Geneva, and Lake Constance. Mussels sampled at a distinct and distant lake in Germany were differentiated from Lake Constance, but less than the Swiss lakes, and genetically clustered close to the Lake Constance populations and River Rhine cluster. The mussel populations from the two basins of Lake Constance (Upper and Lower Lake Constance) and the River Rhine were genetically not differentiated from each other. Previously, weak or no genetic differentiation has been found between European, North American, and Ponto‐Caspian quagga mussel populations (Imo et al., 2010; Marescaux et al., 2016; Therriault et al., 2005). However, all those previous studies relied on fewer genetic markers such as microsatellites, while our study used ddRAD, utilizing high‐resolution markers, which might explain why our study revealed a finer‐scale genetic structure (Le Cam et al., 2020; Luikart et al., 2003; Reitzel et al., 2013). This finding is in line with other studies that demonstrated a higher resolution of population structure using GBS‐like approaches in comparison with microsatellite approaches using only few markers (Ackiss et al., 2020; Sunde et al., 2020). Hence, our study demonstrates the value of ddRAD approaches for studying recent invasions and the quagga mussel system in particularly. It will be exciting to see future studies using RADseq approaches covering the full geographic scale of the quagga mussel invasion.

All the Central European populations investigated revealed a similar and overall high genetic diversity. High genetic diversity has been reported before for quagga mussel populations in Russia, Europe, North America, and also in the native range the Ponto‐Caspian region (Imo et al., 2010; Marescaux et al., 2016; Marshall & Stepien, 2021; Stepien et al., 2002; Therriault et al., 2005). The observation of generally high genetic diversity of quagga mussel population has led to the suggestion that invasion events have occurred without bottleneck or strong founder events. Specifically, it has been suggested that quagga mussel invasion progressed with multiple introductions, and/or strong gene flow between genetically differentiated introduction sites (Marescaux et al., 2016; Marshall & Stepien, 2021). Given the life history traits of the quagga mussel (high fecundity and dispersing pelagic larvae), it is likely a species capable of a high propagule pressure (number of individuals that are introduced to found a population). Propagule pressure has been identified as a key predictor of invasion success (Cassey et al., 2018; Lockwood et al., 2005) and evidence of multiple introductions and a high propagule load have been associated with increased genetic diversity in invasive species (Dlugosch et al., 2015; Dlugosch & Parker, 2008; Viard et al., 2016).

While genetic diversity showed to be quite similar across our sampling sites, effective population sizes varied slightly between the water bodies. Effective population sizes were large in most water bodies except in Lake Geneva and in the German lake. Infinite *N*_e_ or confidence intervals (reported as negative values by the linkage disequilibrium method implemented in NeEstimator) can be interpreted as extremely large population sizes (Do et al., 2014; Gilbert & Whitlock, 2015). However, they also indicate a limiting sample size, not sufficient to estimate the actual population size value. Similarly, the low *N*_e_ detected for Lake Geneva (compared to Lake Neuchâtel or Constance, despite a similar genetic diversity and differentiation) might have been caused by a smaller sample size (Lake Geneva: *N* = 19, Lake Constance: *N* = 28, Lake Neuchâtel: *N* = 25). Overall, our estimates of *N*_e_ based on a limited sample size should only be taken as a first indication and will need to be refined in future studies that allow to increase the sample size or make use of temporal samplings (Gilbert & Whitlock, 2015; Wang, 2016; Waples, 2016). However, taken together the tentative large effective population sizes and high genetic diversity estimates suggest that water bodies investigated in this study have well‐established mussel populations, and potential founder effects have either vanished or were neglectable.

### 4.2 Dispersal scenarios across Swiss lakes suggest invasion from differentiated source populations

Our observation of high genetic diversity across and genetic differentiation between lakes Geneva, Neuchâtel, and Constance has valuable information for two possible dispersal scenarios. One scenario suggests three independent arrivals (into each of the three lakes); each one originating from a distinct and already differentiated source population or region. There is no natural opportunity for colonization via downstream dispersal between these lakes, which all are fed by a different catchment and drainage area: Lake Geneva receives its water from the Rhone catchment, Lake Constance from the Rhine, and Lake Neuchâtel is situated within the Aar River system, which eventually drains downstream in the Rhine River but way downstream of Lake Constance. River Rhine samples are not differentiated from Lake Constance populations, which indicates strong downstream dispersal and establishment of Lake Constance mussels in the Rhine over more than 120 km downstream. As Lake Constance mussels appear to be little differentiated from the German population, it is likely that Lake Constance and the German population have their origin in the same invasion front, while the highly differentiated populations in Lake Geneva and Lake Neuchâtel have rather been sourced from different populations. To unravel whole dispersal pathways, more potential origin populations need to be sampled, for example, France, Netherlands, and North America, including the native range in the Ponto‐Caspian region.

Alternatively, overland transport, for example, via recreational boat transport has been hypothesized a potential way of spreading across Switzerland (De Ventura et al., 2016). De Ventura et al. (2016) showed potential invasion routes via boat transport between lakes Geneva, Constance, and Neuchâtel, and to a lesser extend from lakes in neighbouring countries. As we found similar genetic diversity across populations in those Swiss lakes, but high differentiation between them, our data do not support the hypothesis that these lakes were colonized by overland transport from one lake to the other. Broadcast spawners like dreissenids are unlikely prone to genetic drift due to large (effective) population sizes, but might potentially evolve differentiation between lakes rapidly facilitated by random mating, no reproductive isolation, and random dispersal within lakes (see discussion below about lack of population structure and differentiation within a lake) and geographic separation between lakes. As quagga mussels were first discovered in Lake Geneva in 2015 and in Lake Constance in 2016 (Haltiner et al., 2022), differentiation between those populations would have needed to be very rapid, that is, within 4 years in Lake Geneva, and 3 years in Lake Constance (the samples for this study were taken in 2019). Even if we assume that first detection only happened a few years after colonization that would be fast given a generation time of 1 year (as assumed in Marescaux et al., 2016). How fast differentiation can evolve between geographically separated lakes in *Dreissena* has not yet been investigated. However, differentiation over time has been examined in Lake Erie (border lake between the USA and Canada). Marshall and Stepien (2021) followed the Lake Erie quagga population over 18 years using 10 microsatellite markers and showed that the allelic composition shifted significantly between 1998 and 2016, in a time span of 18 years, but not in shorter time spans they evaluated (between 1998 and 2011: 11 years; between 2011 and 2016: 5 years). Given these observations from Lake Erie, a substantial differentiation across Swiss lakes in just 5 years seems unlikely. In summary, our results demonstrate the potential and value of high‐resolution markers for fine‐scale population structure studies to provide insights on the dispersal of invasive species across lakes. Such studies are relevant as they help to understand the dispersal routes, which is essential to eliminate invasion pathways into other lakes in the future. But it will be important to extend such high‐resolution markers approaches to the full range of the biological invasion as well as the native range of quagga mussels to conclusively comment on the colonization history of the perialpine lakes.

### 4.3 No signs for local adaptation to depth or reproductive isolation within Lake Constance

Between sites or along a depth gradient, several potential barriers (e.g., thermocline, currents) can create population structure or isolation by distance. Within Lake Constance, quagga mussel populations revealed no population structure and there was no detectable genetic differentiation across populations from different sampling sites or depth (all pairwise *F*_st_ < 0). The observed lack of any population genetic patterns is comparable to patterns observed for a range of marine broadcast spawners (reviewed in Pascual et al., 2017). In general, their longer planktonic larval phase allows long‐distance dispersal and can hinder the evolution of population differentiation and prevent signals of isolation by distance (e.g., Charrier et al., 2006; Pascual et al., 2017). In particular, marine species with high fecundity, large population sizes, and larvae with long‐distance dispersal can show low genetic structures (Palumbi, 1994). Broadcast spawners in freshwater like the quagga mussel are characterized by high genetic variability (Brown & Stepien, 2010; Imo et al., 2010; Marescaux et al., 2016; Stepien et al., 2002; Therriault et al., 2005), high fecundity, and long planktonic larva phase of up to a month or longer (Karatayev & Burlakova, 2022). This fits well with our observations of the quagga mussels in Lake Constance, which show a genetic diversity and effective population size indicative of a large established population and no differentiation between populations from different sampling sites within the lake. However, it has been shown in marine systems that life history characteristics are not the only predictor of dispersal distance but that there are other barriers to gene flow such as for example habitats (Kelly & Palumbi, 2010). For the quagga mussels in Lake Constance, no such barriers seem to exist or currents within the lake are not stable enough to create consistent patterns of larval distribution that resulted in a detectable population structure.

To establish a new population and spread within a deep lake like Lake Constance, quagga mussels need to be able to cope with different habitat sites and depths. Despite the absence of any population structure within Lake Constance, we observed phenotypic variation across quagga mussels regarding shell coloration. This might suggest a correlation of shell morphology with depth (Dermott & Munawar, 1993), which could indicate either a plastic change or local adaptation. Given that mussels in Lake Constance spread into the deeper areas within a few years (Haltiner et al., 2022), this would require a rather rapid adaptation, which might have been facilitated by standing genetic variation or colonizing mussels might have been pre‐adapted to different depths. Our scans of the genome identified two candidate loci potentially shaped by selection. However, those loci are weak candidates for indicating local adaptation to depth, as allele frequency changes at these loci are only moderated across depths (appr. 14%) and comparable to changes between sampling sites (also appr. 14%). This suggests that differentiation at those loci might well be the result of neutral stochastic drift effects. Furthermore, it might be that the scan rather picked up a signature of global and not local selection, or that the scan was limited due to non‐equilibrium demographic history in an expanding invasive population (Booker et al., 2021; Lotterhos & Whitlock, 2015). These might be the most relevant concerns causing caution in interpreting outlier loci detected for our study (Bierne et al., 2011; Booker et al., 2020); however, others have raised concerns regarding the limited power and probability of detecting associated loci using RADseq (Catchen et al., 2017; Lowry et al., 2017).

To overcome the plenty shortcomings of relying on molecular signatures to identify local adaptation, we also conducted a reciprocal transplant experiment, also known as the gold‐standard experiment to detect local adaptation (Savolainen et al., 2013). However, in line with the genome scan, the reciprocal transplant experiment did also not provide much evidence for local adaptation to living in the deep. Mussels grew slower in deep than in shallow habitats but irrespective of their depth of origin. Survival of mussels was also not depended on the origin of the mussel. Hence, both fitness proxies: shell growth and survival showed no signs of local adaptation. It might be worth noting that our growth estimates were population estimates as individuals were not individually marked. Hence, our analysis could have been affected by a differential survival of larger versus smaller individual. Quagga mussels have a typical lifetime of 4–5 years (with estimated longevity up to 30 years in the profundal zone) and can grow up to about 4.7 cm (Karatayev & Burlakova, 2022). In our experiment, we used mussels between 8 and 16 mm. It is hence unlikely that age mortality had a significant effect in our 100‐day experiment. Furthermore, we could demonstrate (see Figure S1) that growth was independent of density in the bags.

Taking together the outcome of our genome scan and reciprocal transplant experiment, it is likely that the spreading and establishment of quagga mussels into the deeper lake areas was facilitated by plastic responses to all kind of different conditions in the lake. Earlier studies revealed similar results, and across various mollusc taxa, it has been shown that they are highly plastic, especially in shell morphology and colour (e.g., for clams, Jokela & Mutikainen, 1995; e.g., for mussels, Leonard et al., 1999; review for gastropods, Whelan, 2021). Furthermore, previous studies (using limited marker sets) have detected no genetic differences between the morphologically distinct mussels and suggested that the deep living morph is rather the results of plasticity than local adaptation (Claxton et al., 1998; Spidle et al., 1994; Stepien et al., 1999, 2002). A common garden experiment by Peyer et al. (2010) also suggests plasticity as the cause of differences between shell morphs. Therefore, our work supports the hypothesis that quagga mussels' high phenotypic plasticity and life history traits, being a broadcast spawner with high fecundity and dispersing pelagic larvae, facilitate their fast dispersal and spread in a lake across many habitat types.

## 5 CONCLUSION

This study provides first genetic insights into potential dispersal pathways of quagga mussels across perialpine lakes in Switzerland. We show that quagga mussel populations across Swiss lakes are well‐established populations with a high genetic diversity and effective population sizes. This suggests that populations across Swiss lakes likely have been colonized independently from different source populations. While overland transport of recreational boats seems to have not left any genetic traces of gene flow across Swiss lakes, the high genetic diversity we detected across those only recently colonized lakes suggests that lakes were founded by a high propagule pressure potentially by multiple introductions. Although our study has its sampling constrains (especially outside of Switzerland) and hence provides only limited details on the colonization pathways into Swiss lakes, it demonstrates the value and potential of genetic studies using high‐resolution marker sets in the system. This is especially highlighted by our findings, based on an extensive sampling effort within Lake Constance, which suggests that quagga mussels' phenotypic plasticity might be a relevant characteristic that makes quagga mussels such successful invaders. For now, mussels seem to disperse randomly across Lake Constance and do not show any populations structure nor genetic differentiation or local adaptation along depth. Based on our findings, we suggest to implement high‐resolution genetic approaches into future surveys of quagga mussels for monitoring purposes and to determine if and which management interventions are effective.

## CONFLICT OF INTEREST STATEMENT

There is no conflict of interest.

## ETHICS STATEMENT

The manuscript has not been submitted elsewhere, and all research meets the ethical guidelines of Switzerland.

## Supporting information


Appendix S1.

## Data Availability

Raw data underlying this article are available at the data repository ERIC https://doi.org/10.25678/0007EG, and raw sequence reads used to generate the results of this study are available at the European Nucleotide Archive (PRJEB70393).
