# Supplementary material for: Population genetic insights into establishment, adaptation, and dispersal of the invasive quagga mussel across perialpine lakes
Source: Evol Appl. 2023 Dec 8;17(1):e13620. doi: 10.1111/eva.13620 (PMC10809192; doi:10.1111/eva.13620)
Supplement: Supplementary file 1 — Appendix S1. [file EVA-17-e13620-s001.docx]

Population genetic insights into establishment, adaptation, and dispersal of the invasive quagga mussel across perialpine lakes

Linda Haltiner ^1,2^, Piet Spaak ^1,2^, Stuart R Dennis ^1,3^, Philine G. D. Feulner ^4,5^

^1^ Aquatic Ecology, Swiss Federal Institute of Aquatic Science and Technology (Eawag), Dübendorf, Switzerland

^2^ Environmental Systems Sciences, ETH Zürich, Zürich, Switzerland

^3^ current address: Dept. IT services, Swiss Federal Institute of Aquatic Science and Technology (Eawag), Dübendorf, Switzerland

^4^ Fish Ecology and Evolution, Center for Ecology, Evolution and Biogeochemistry, Swiss Federal Institute of Aquatic Science and Technology (Eawag), Kastanienbaum, Switzerland

^5^ Aquatic Ecology, Institute of Ecology and Evolution, University of Bern, Bern, Switzerland

# Data availability

Raw data underlying this article will be available on the Eawag data repository ERIC [https://doi.org/10.25678/0007EG](https://doi.org/10.25678/0007DF) and raw sequence reads used to generate the results of this study are available on a short read archive SRA.

# Supplementary material


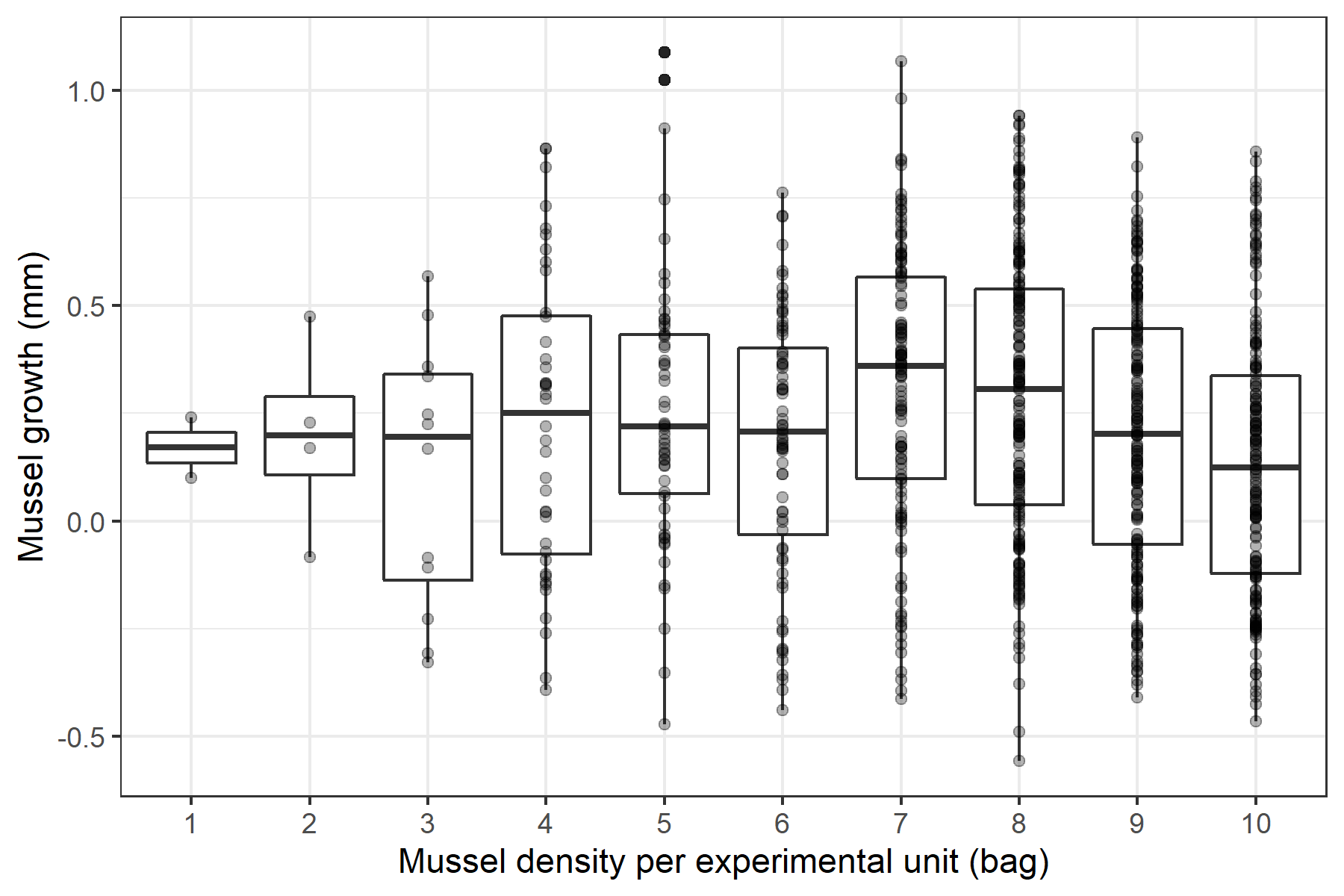


**Figure S1** No density dependent growth of mussels in the reciprocal transplant experiment. We show number of individuals per bag plotted against the mean growth (individual size at the end of the experiment – mean sizes at the start of the experiment). The variance and the mean growth did not change as the density increases. This indicates that density (i.e., competition for resources) did not influence the growth in our experiment.


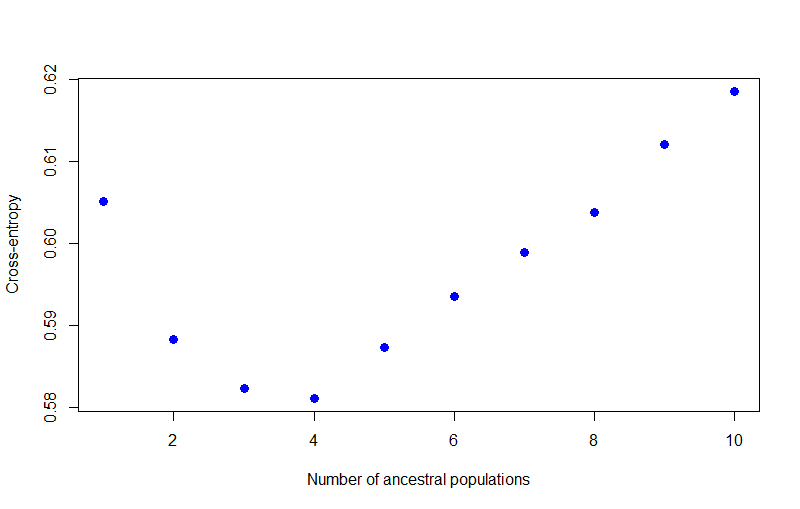


**Figure S2** Cross-entropy values for the admixture analysis (LEA) were lowest at K = 4 for the across Swiss lake data set. For the admixture analysis, we used K = 4 (Figure 2) but we also show K = 5 in Figure S3 representing five geographic ranges (lakes Geneva, Neuchâtel, Germany, Rhine, Lake Constance)


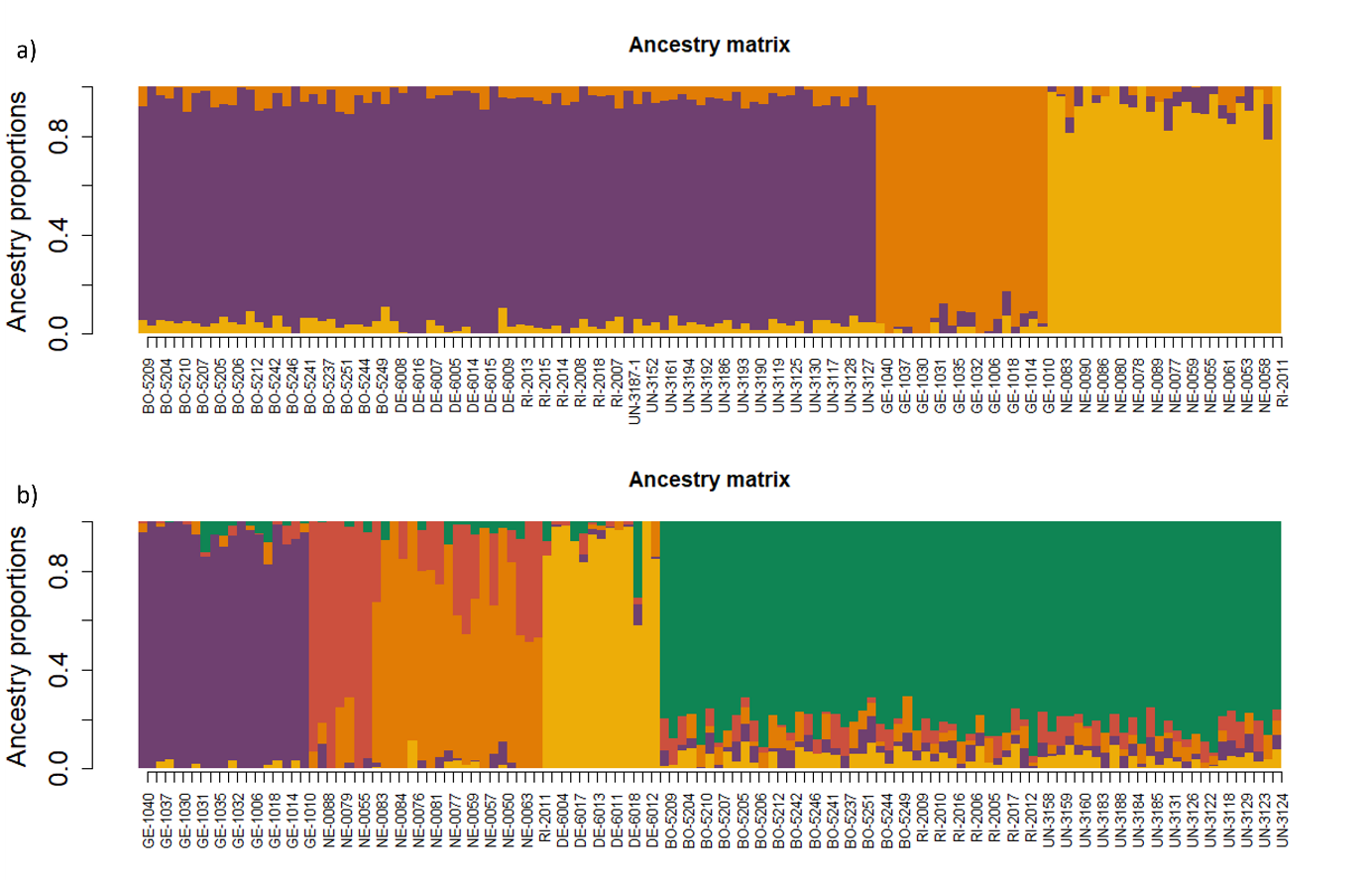


**Figure S3** Quagga mussels’ genetic composition (admixture proportions estimated by LEA) across Swiss lakes with K = 3 (a) and K= 5 (b). K = 5 instead of K = 4 shows different ancestry proportions for mussels from Lake Neuchâtel, but the mussels from Lake Constance and the River Rhine still show similar ancestry proportions. Each bar represents an individual and the colours represent the proportion of each of the five genetic contributions. GE = mussels from Lake Geneva, NE = Lake Neuchâtel, DE = Germany, RI = Rhine, BO = Upper Lake Constance, UN = Lower Lake Constance


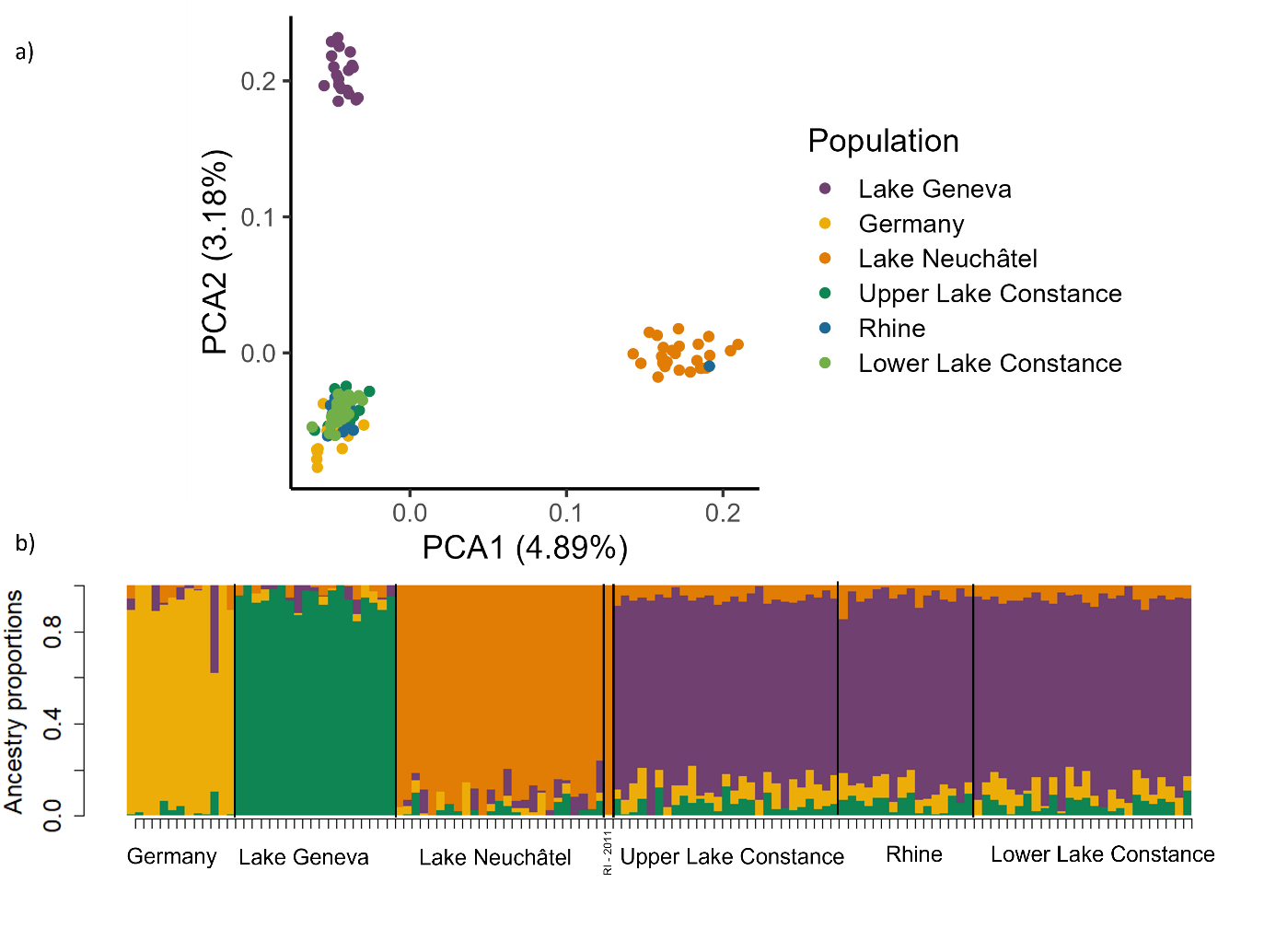


**Figure S4** PCA (a) and admixture analysis (b) for thinned data set (14,300 SNPs) across Swiss lakes. Results were overall consistent with the those from the full dataset.


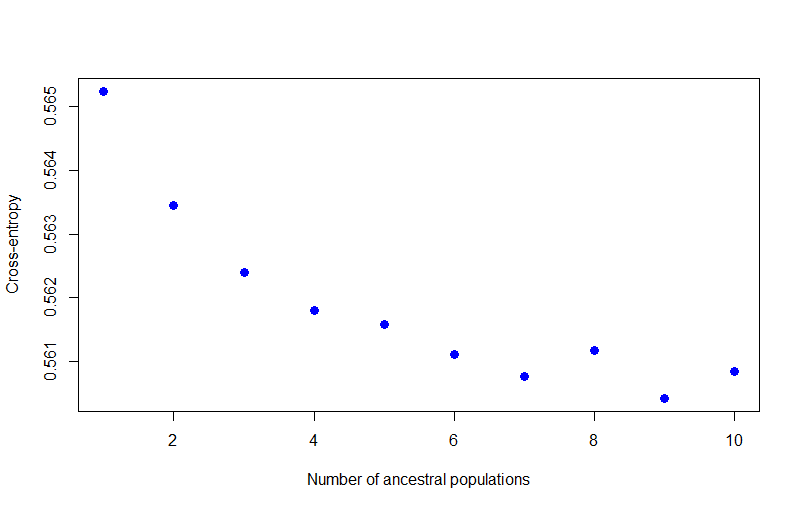


**Figure S5** Cross-entropy values for the admixture analysis (LEA) were lowest at K = 9 for the within Lake Constance data set. For the admixture analysis, we used K = 3 (Figure 3) representing our three depth categories (10, 30, 60 m). We also show K = 9 in Figure S5.


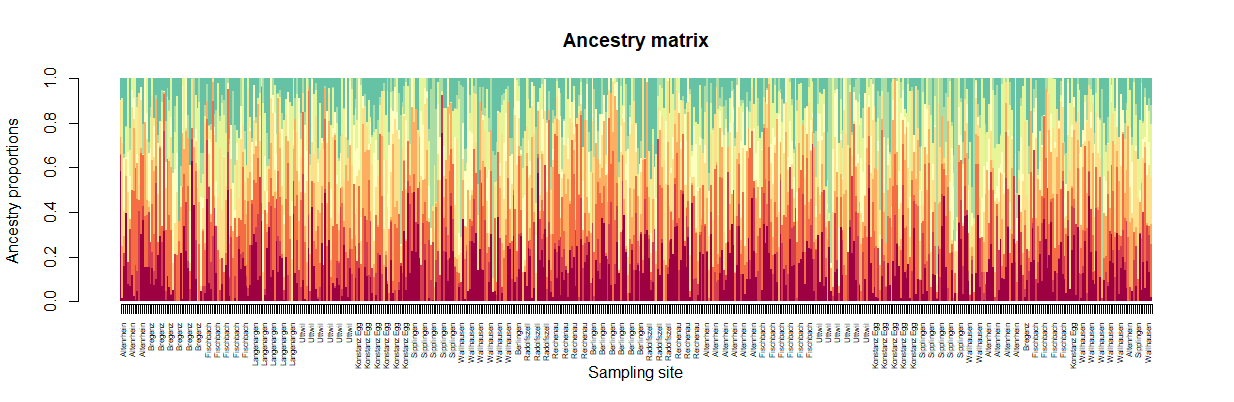


**Figure S6** Quagga mussels’ genetic composition (admixture proportions estimated by LEA) within Lake Constance with K = 9. K = 9 instead of K = 3 still shows similar ancestry proportions across all sampling sites. Each bar represents an individual (ordered by sampling site) and the colours represent the proportion of each of the nine genetic contributions.


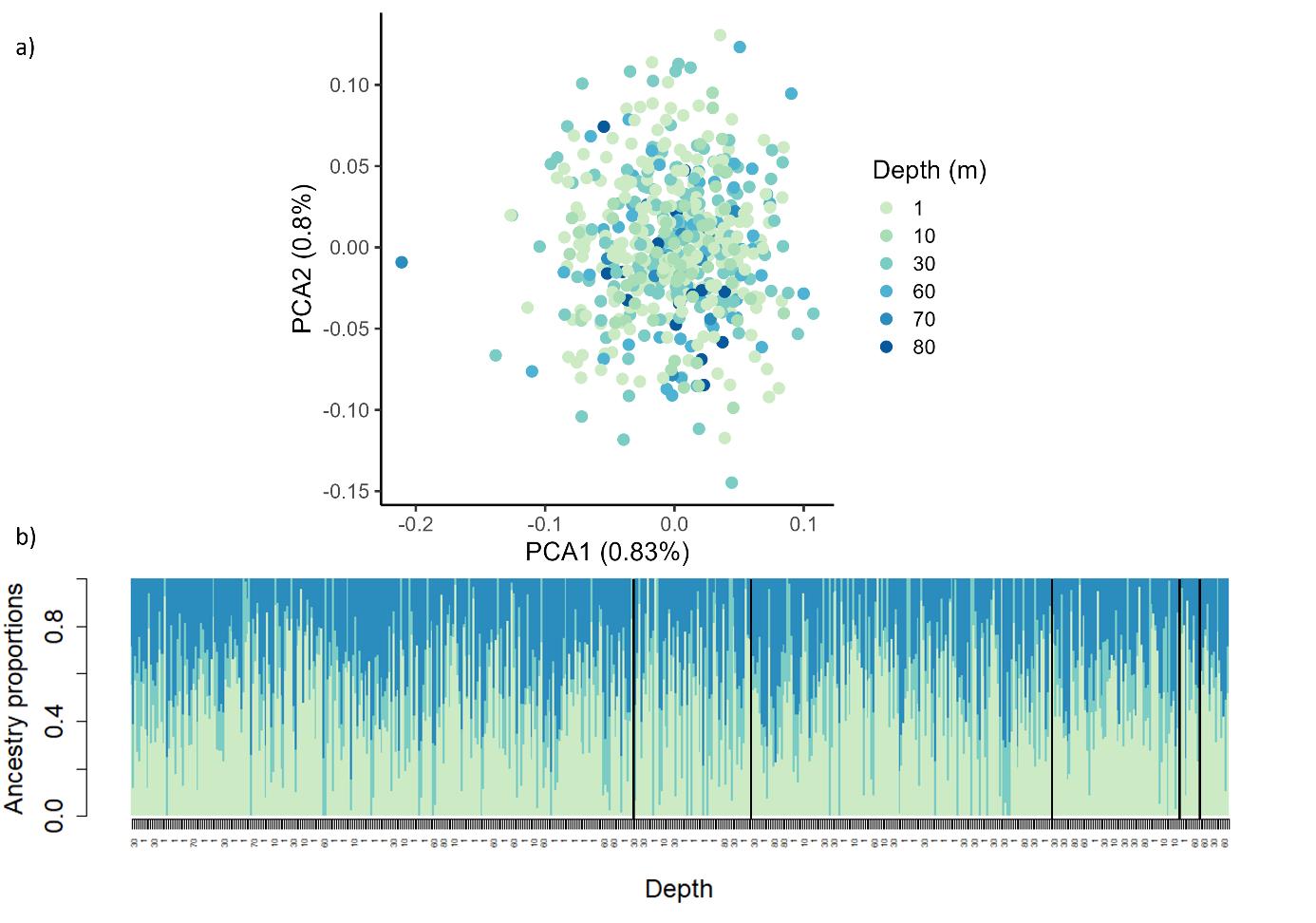


**Figure S7** PCA (a) and admixture analysis (b) for thinned data set (796 SNPs) within Lake Constance. Results were overall consistent with the those from the full dataset.


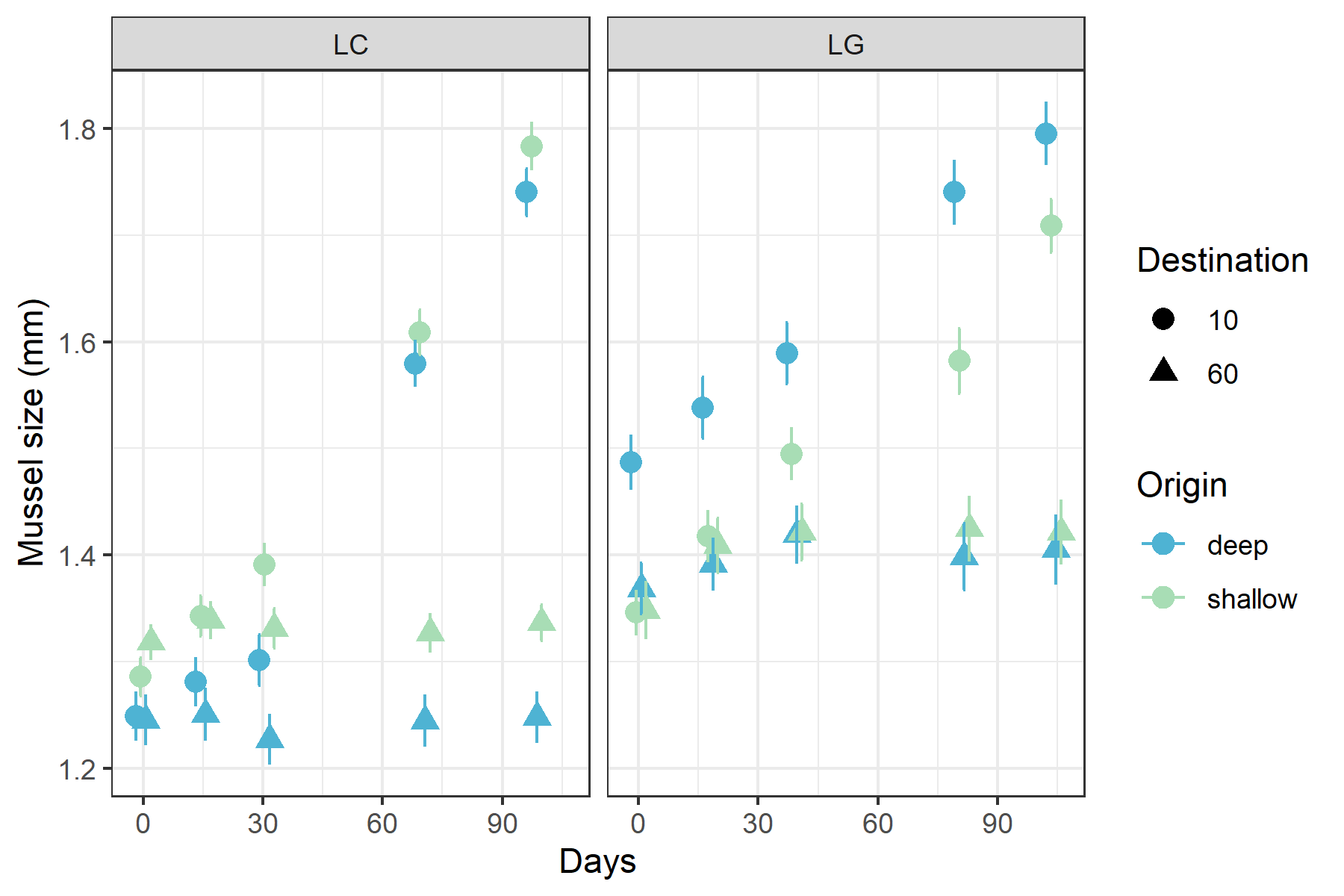


**Figure S8** Mussel grew larger in 10 m than raised in 60 m. Mean mussel sizes per experimental unit (mean ± SE) are shown over time for Lake Constance (LC, left) and Lake Geneva (LG, right). Shapes indicate depth of destination (points = 10 m, triangle = 60 m) and colours display the depth of origin of the mussels (light blue for mussels from 60 m, light green for mussels from 10 m).

**Table S1** Sampling sites in Lake Constance. At each sampling site, mussels along two depth transects were collected with an Ekman grab sampler. Letters correspond to sampling sites in Figure 1. Sample size refers to the number of individuals genotyped and used in population genetic analyses.

| **Lake** | **Lake basin** | **Town** | **Site** | **Coordinates N** | **Coordinates E** | **Nr of transects** | **Sampling depths** | **Label in Figure 1** | **Sample size N** |
| --- | --- | --- | --- | --- | --- | --- | --- | --- | --- |
| Lake Constance | Upper Lake | Altenrhein | Strandbad | 47° 29′ 37.9" | 9° 33′ 1.6" | 2 | 1,30,60,80 | a | 71 |
| Lake Constance | Upper Lake | Bregenz | Seecamping | 47° 30′ 26.6" | 9° 42′ 59.2" | 2 | 1,30,60 | b | 33 |
| Lake Constance | Upper Lake | Fischbach | Freibad | 47° 39' 58.5'' | 9° 25' 03.8'' | 2 | 1,30,60 | d | 76 |
| Lake Constance | Upper Lake | Langenargen | Malereck | 47° 35′ 22.0" | 9° 32′ 54.2" | 2 | 1,60 | c | 28 |
| Lake Constance | Upper Lake | Uttwil | Hafen | 47° 35' 07'' | 09° 20' 50'' | 2 | 1,30 | e | 57 |
| Lake Constance | Lake Überlingen | Konstanz Egg | Pumphaus | 47° 41′ 30.4" | 9° 12′ 6.7" | 2 | 1,30,60 | f | 57 |
| Lake Constance | Lake Überlingen | Sipplingen | Seestrasse | 47° 47′ 28.1" | 9° 06′ 31.8" | 2 | 1,30,80 | g | 52 |
| Lake Constance | Lake Überlingen | Wallhausen | Teufesltisch | 47° 45′ 11.64" | 9° 07′ 31.3" | 2 | 1,30,60,70,80 | h | 73 |
| Lake Constance | Lower Lake | Berlingen | Parkplatz, westlich Ortseingang | 47° 40' 22.1'' | 9° 00' 38.9'' | 2 | 1,10 | k | 38 |
| Lake Constance | Lower Lake | Radolfszell | Bahnhof | 47° 44' 08" | 08° 58' 02" | 2 | 1,10 | j | 29 |
| Lake Constance | Lower Lake | Reichenau | Westlich Schiffslände und Hafen | 47° 41′ 25.5" | 9° 03′ 11.5" | 2 | 1,10 | i | 35 |

**Table S2** Information of the individuals used in the across Swiss lakes data set. This is a subset of the data and the whole list can be found here: https://doi.org/10.25678/0007EG

| **Sample_id** | **used in within Lake Constance analysis** | **Library** | **ng DNA** | **Lake basin** | **Site** | **Transect** | **Depth** | **Length  (mm)** | **Height  (mm)** | **Width  (mm)** | **Phenotype** | **Reads** | **Mapped  (%)** | **Retained reads after mapping** |
| --- | --- | --- | --- | --- | --- | --- | --- | --- | --- | --- | --- | --- | --- | --- |
| BO-5200 | x | 12A | 200 | Obersee | Uttwil | 2 | 1 | 15.31 | 8.55 | 5.72 | s | 7’010’405 | 96.56 | 6’769’247 |
| BO-5202 | x | 13B | 200 | Obersee | Uttwil | 2 | 1 | 14.77 | 8.28 | 5.93 | s | 1’932’460 | 96.7 | 1’868’689 |
| BO-5203 | x | 14A | 200 | Obersee | Uttwil | 2 | 1 | 15.08 | 8.73 | 6.31 | s | 4’369’008 | 96.74 | 4’226’578 |
| BO-5204 | x | 14B | 200 | Obersee | Uttwil | 2 | 1 | 14.68 | 8.41 | 6.97 | s | 1’370’991 | 96.48 | 1’322’732 |
| BO-5205 | x | 15B | 200 | Obersee | Uttwil | 2 | 1 | 13.86 | 7.78 | 5.67 | s | 2’640’282 | 96.68 | 2’552’625 |
| BO-5206 | x | 16A | 200 | Obersee | Uttwil | 2 | 1 | 13.65 | 7.68 | 5.12 | s | 4’262’346 | 96.71 | 4’122’115 |
| BO-5207 | x | 17A | 200 | Obersee | Uttwil | 2 | 1 | 12.29 | 7.01 | 5.9 | s | 4’365’906 | 96.66 | 4’220’085 |
| BO-5208 | x | 17B | 200 | Obersee | Uttwil | 2 | 1 | 14.42 | 8.06 | 5.25 | s | 2’941’113 | 96.36 | 2’834’056 |
| BO-5209 | x | low | 65.5 | Obersee | Uttwil | 2 | 1 | 15.94 | 9.01 | 6.52 | s | 3’811’748 | 95.99 | 3’658’897 |
| BO-5210 | x | 11A | 200 | Obersee | Uttwil | 2 | 1 | 14.41 | 8.68 | 5.66 | s | 5’176’175 | 96.63 | 5’001’738 |
| BO-5211 | x | 11B | 200 | Obersee | Uttwil | 2 | 1 | 14.6 | 7.88 | 5.18 | s | 1’885’966 | 96.64 | 1’822’598 |
| BO-5212 | x | 12B | 200 | Obersee | Uttwil | 2 | 1 | 12.99 | 7.67 | 5.89 | s | 2’623’558 | 96.77 | 2’538’817 |
| BO-5213 | x | 13A | 200 | Obersee | Uttwil | 2 | 1 | 12.94 | 7.48 | 5.41 | s | 4’833’156 | 96.67 | 4’672’212 |
| BO-5236 | x | 15B | 200 | Obersee | Altenrhein | 2 | 1 | 8.92 | 5.47 | 3.24 | s | 5’954’504 | 96.72 | 5’759’196 |

**Table S3** Information of the individuals sampled within Lake Constance. This is a subset of the data and the whole list can be found here: https://doi.org/10.25678/0007EG

| **Sample_id** | **Library** | **ng DNA** | **Lake basin** | **Site** | **Transect** | **Depth** | **Length  (mm)** | **Height  (mm)** | **Width  (mm)** | **Phenotype** | **Reads** | **Mapped  (%)** | **Retained reads after mapping** |
| --- | --- | --- | --- | --- | --- | --- | --- | --- | --- | --- | --- | --- | --- |
| BO-5200 | 12A | 200 | Obersee | Uttwil | 2 | 1 | 15.31 | 8.55 | 5.72 | s | 7’010’405.00 | 96.56 | 6’769’247.07 |
| BO-5202 | 13B | 200 | Obersee | Uttwil | 2 | 1 | 14.77 | 8.28 | 5.93 | s | 1’932’460.00 | 96.7 | 1’868’688.82 |
| BO-5203 | 14A | 200 | Obersee | Uttwil | 2 | 1 | 15.08 | 8.73 | 6.31 | s | 4’369’008.00 | 96.74 | 4’226’578.34 |
| BO-5204 | 14B | 200 | Obersee | Uttwil | 2 | 1 | 14.68 | 8.41 | 6.97 | s | 1’370’991.00 | 96.48 | 1’322’732.12 |
| BO-5205 | 15B | 200 | Obersee | Uttwil | 2 | 1 | 13.86 | 7.78 | 5.67 | s | 2’640’282.00 | 96.68 | 2’552’624.64 |
| BO-5206 | 16A | 200 | Obersee | Uttwil | 2 | 1 | 13.65 | 7.68 | 5.12 | s | 4’262’346.00 | 96.71 | 4’122’114.82 |
| BO-5207 | 17A | 200 | Obersee | Uttwil | 2 | 1 | 12.29 | 7.01 | 5.9 | s | 4’365’906.00 | 96.66 | 4’220’084.74 |
| BO-5208 | 17B | 200 | Obersee | Uttwil | 2 | 1 | 14.42 | 8.06 | 5.25 | s | 2’941’113.00 | 96.36 | 2’834’056.49 |
| BO-5209 | low | 65.5 | Obersee | Uttwil | 2 | 1 | 15.94 | 9.01 | 6.52 | s | 3’811’748.00 | 95.99 | 3’658’896.91 |
| BO-5210 | 11A | 200 | Obersee | Uttwil | 2 | 1 | 14.41 | 8.68 | 5.66 | s | 5’176’175.00 | 96.63 | 5’001’737.90 |
| BO-5211 | 11B | 200 | Obersee | Uttwil | 2 | 1 | 14.6 | 7.88 | 5.18 | s | 1’885’966.00 | 96.64 | 1’822’597.54 |
| BO-5212 | 12B | 200 | Obersee | Uttwil | 2 | 1 | 12.99 | 7.67 | 5.89 | s | 2’623’558.00 | 96.77 | 2’538’817.08 |
| BO-5213 | 13A | 200 | Obersee | Uttwil | 2 | 1 | 12.94 | 7.48 | 5.41 | s | 4’833’156.00 | 96.67 | 4’672’211.91 |
| BO-5236 | 15B | 200 | Obersee | Altenrhein | 2 | 1 | 8.92 | 5.47 | 3.24 | s | 5’954’504.00 | 96.72 | 5’759’196 |

**Table S4** SNP filtering protocol followed after the dDocent pipeline (Puritz et al. 2014). Steps in colour indicate different filter values between the two data sets (across Swiss lakes and within Lake Constance).

|  | | | **data set: across Swiss lakes** | | | | | **data set: within Lake Constance** | | | | |
| --- | --- | --- | --- | --- | --- | --- | --- | --- | --- | --- | --- | --- |
| **Packages** | **Command and values** | **Filter explanation** | **Nr of individuals started** | **Nr of individuals reatained** | **Nr of variants started** | **Nr of variants retained** | **variants lost  (%)** | **Nr of individuals started** | **Nr of individuals reatained** | **Nr of variants started** | **Nr of variants retained** | **variants lost  (%)** |
| vcftools | --max-missing 0.5 -- mac 3 --minQ 30 | Remove sites with high missingness across individuals, quality scores and minor allele count | 158 | 158 | 10’576’951 | 2’713’846 | 74.3 | 597 | 597 | 16’614’375 | 3’477’612 | 79.1 |
| vcftools | --minDP 3 | Minimum read depth | 158 | 158 | 2’713’846 | 2’713’846 | 0.0 | 597 | 597 | 3’477’612 | 3’477’612 | 0.0 |
| vcftools makw | --missing-indv mawk '$5 > 0.7' out.imiss  --remove lowDP.indv | Remove individuals with high missingness across sites | 158 | 143 | 2’713’846 | 2’713’846 | 0.0 |  |  |  |  |  |
|  | -missing-indv mawk '$5 > 0.85' out.imiss  --remove lowDP.indv | within Lake Constance: remove individuals with high missingness across sites |  |  |  |  |  | 597 | 565 | 3’477’612 | 3’477’612 | 0.0 |
| vcftools | --max-missing 0.1 --maf 0.01 --meanDP 10 | More stringent missingness filter, minor allele frequency and mean depth filter | 143 | 143 | 2’713’846 | 468’436 | 82.7 | 565 | 565 | 3’477’612 | 290’743 | 91.6 |
| vcffilter | -s -f "AB > 0.25 & AB < 0.75 \| AB < 0.01" | Allele balance filter | 143 | 143 | 468’436 | 353’427 | 24.6 | 565 | 565 | 290’743 | 21’639 | 92.6 |
| vcffilter | -f "SAF / SAR > 100 & SRF / SRR > 100 \| SAR / SAF > 100 & SRR / SRF > 100" | Filter out sites which have reads from both forward and reverse reads | 143 | 143 | 353’427 | 305’283 | 13.6 | 565 | 565 | 21’639 | 18’521 | 14.4 |
| vcffilter | -f "MQM / MQMR > 0.9 & MQM / MQMR < 1.05" | Filter for mapping quality between reference and alternate alleles | 143 | 143 | 305’283 | 253’905 | 16.8 | 565 | 565 | 18’521 | 15’660 | 15.4 |
| vcffilter | -f "PAIRED > 0.05 & PAIREDR > 0.05 & PAIREDR / PAIRED < 1.75 & PAIREDR / PAIRED > 0.25 \| PAIRED < 0.05 & PAIREDR < 0.05" -s | Filter for discrepancy in the paired statis of the reads and the reference or alternate alleles | 143 | 143 | 253’905 | 248’476 | 2.1 | 565 | 565 | 15’660 | 15’420 | 1.5 |
| vcffilter | -f "QUAL / DP > 0.25" | Filter for ratio of quality score to depth | 143 | 143 | 248’476 | 228’620 | 8.0 | 565 | 565 | 15’420 | 14’193 | 8.0 |
| vcftools | --site-depth  --exclude-positions --max-meanDP50 | Filter for maximum mean depth | 143 | 143 | 228’620 | 205’583 | 10.1 |  |  |  |  |  |
| vcftools | --site-depth  --exclude-positions --max-meanDP40 | within Lake Constance: filter for maximum mean depth |  |  |  |  |  | 565 | 565 | 14’193 | 12’608 | 11.2 |
| vcftools | --remove-indels | Remove indels | 143 | 143 | 205’583 | 187’982 | 8.6 | 565 | 565 | 12’608 | 11’573 | 8.2 |
| vcftools | --max-missing 0.95 | more stringent missingness filter | 143 | 143 | 187’982 | 83’642 | 55.5 |  |  |  |  |  |
| vcftools | --max-missing 0.93 | within Lake Constance: more stringent missingness filter |  |  |  |  |  | 565 | 565 | 11’573 | 5’142 | 55.6 |
| vcftools | --min-alleles 2   --max-alleles 2 | Keep only biallelic sites. | 143 | 143 | 83’642 | 81’197 | 2.9 | 565 | 565 | 5’142 | 4’939 | 3.9 |
|  |  | Both data sets still contain 16 identical individuals to calculate genotyping error. They are removed after the calculations. | 143 | 127 | 81’197 | 81’197 |  | 565 | 549 | 4’939 | 4’939 |  |

**Table S5** List of outlier loci potentially indicating signatures of adaptive evolution. Reported are the scaffold and position of 59 outlier loci, the two respective alleles and their frequencies at each of the three depth categories.

| **Scaffold** | **Position** | **Allele 1** | **Frequency allele 1** | **Allele 2** | **Frequency allele 2** | **Depth** |
| --- | --- | --- | --- | --- | --- | --- |
| VMBQ01000016.1 | 318019 | T | 0.3547 | C | 0.6453 | 1 |
| VMBQ01000016.1 | 318019 | T | 0.4021 | C | 0.5979 | 30 |
| VMBQ01000016.1 | 318019 | T | 0.4419 | C | 0.5581 | 60 |
| VMBQ01000016.1 | 631278 | T | 0.2840 | C | 0.7160 | 1 |
| VMBQ01000016.1 | 631278 | T | 0.2500 | C | 0.7500 | 30 |
| VMBQ01000016.1 | 631278 | T | 0.1747 | C | 0.8253 | 60 |
| VMBQ01000018.1 | 430090 | A | 0.7787 | C | 0.2213 | 1 |
| VMBQ01000018.1 | 430090 | A | 0.8209 | C | 0.1791 | 30 |
| VMBQ01000018.1 | 430090 | A | 0.8721 | C | 0.1279 | 60 |
| VMBQ01000018.1 | 430212 | G | 0.7623 | A | 0.2377 | 1 |
| VMBQ01000018.1 | 430212 | G | 0.8020 | A | 0.1980 | 30 |
| VMBQ01000018.1 | 430212 | G | 0.8663 | A | 0.1337 | 60 |
| VMBQ01000018.1 | 430216 | G | 0.7623 | A | 0.2377 | 1 |
| VMBQ01000018.1 | 430216 | G | 0.8020 | A | 0.1980 | 30 |
| VMBQ01000018.1 | 430216 | G | 0.8663 | A | 0.1337 | 60 |
| VMBQ01000018.1 | 430305 | A | 0.7685 | T | 0.2315 | 1 |
| VMBQ01000018.1 | 430305 | A | 0.7925 | T | 0.2075 | 30 |
| VMBQ01000018.1 | 430305 | A | 0.8706 | T | 0.1294 | 60 |
| VMBQ01000023.1 | 213795 | C | 0.7126 | T | 0.2874 | 1 |
| VMBQ01000023.1 | 213795 | C | 0.7724 | T | 0.2276 | 30 |
| VMBQ01000023.1 | 213795 | C | 0.7849 | T | 0.2151 | 60 |
| VMBQ01000030.1 | 134084 | A | 0.6786 | T | 0.3214 | 1 |
| VMBQ01000030.1 | 134084 | A | 0.7063 | T | 0.2937 | 30 |
| VMBQ01000030.1 | 134084 | A | 0.7841 | T | 0.2159 | 60 |
| VMBQ01000030.1 | 134290 | C | 0.6799 | A | 0.3201 | 1 |
| VMBQ01000030.1 | 134290 | C | 0.6993 | A | 0.3007 | 30 |
| VMBQ01000030.1 | 134290 | C | 0.7874 | A | 0.2126 | 60 |
| VMBQ01000030.1 | 134299 | T | 0.6765 | A | 0.3235 | 1 |
| VMBQ01000030.1 | 134299 | T | 0.6993 | A | 0.3007 | 30 |
| VMBQ01000030.1 | 134299 | T | 0.7816 | A | 0.2184 | 60 |
| VMBQ01000030.1 | 514625 | T | 0.7243 | C | 0.2757 | 1 |
| VMBQ01000030.1 | 514625 | T | 0.7624 | C | 0.2376 | 30 |
| VMBQ01000030.1 | 514625 | T | 0.8155 | C | 0.1845 | 60 |
| VMBQ01000030.1 | 514914 | A | 0.8151 | T | 0.1849 | 1 |
| VMBQ01000030.1 | 514914 | A | 0.8552 | T | 0.1448 | 30 |
| VMBQ01000030.1 | 514914 | A | 0.9176 | T | 0.0824 | 60 |
| VMBQ01000032.1 | 109062 | G | 0.6474 | T | 0.3526 | 1 |
| VMBQ01000032.1 | 109062 | G | 0.5799 | T | 0.4201 | 30 |
| VMBQ01000032.1 | 109062 | G | 0.5471 | T | 0.4529 | 60 |
| VMBQ01000032.1 | 109171 | A | 0.4213 | G | 0.5787 | 1 |
| VMBQ01000032.1 | 109171 | A | 0.4236 | G | 0.5764 | 30 |
| VMBQ01000032.1 | 109171 | A | 0.3214 | G | 0.6786 | 60 |
| VMBQ01000032.1 | 109179 | A | 0.4178 | G | 0.5822 | 1 |
| VMBQ01000032.1 | 109179 | A | 0.4236 | G | 0.5764 | 30 |
| VMBQ01000032.1 | 109179 | A | 0.3214 | G | 0.6786 | 60 |
| VMBQ01000032.1 | 109322 | G | 0.6458 | T | 0.3542 | 1 |
| VMBQ01000032.1 | 109322 | G | 0.5862 | T | 0.4138 | 30 |
| VMBQ01000032.1 | 109322 | G | 0.5471 | T | 0.4529 | 60 |
| VMBQ01000035.1 | 72441 | G | 0.6986 | A | 0.3014 | 1 |
| VMBQ01000035.1 | 72441 | G | 0.7568 | A | 0.2432 | 30 |
| VMBQ01000035.1 | 72441 | G | 0.7798 | A | 0.2202 | 60 |
| VMBQ01000042.1 | 499914 | T | 0.4155 | G | 0.5845 | 1 |
| VMBQ01000042.1 | 499914 | T | 0.4291 | G | 0.5709 | 30 |
| VMBQ01000042.1 | 499914 | T | 0.5341 | G | 0.4659 | 60 |
| VMBQ01000052.1 | 235396 | A | 0.7801 | T | 0.2199 | 1 |
| VMBQ01000052.1 | 235396 | A | 0.8273 | T | 0.1727 | 30 |
| VMBQ01000052.1 | 235396 | A | 0.8571 | T | 0.1429 | 60 |
| VMBQ01000052.1 | 235418 | A | 0.7818 | G | 0.2182 | 1 |
| VMBQ01000052.1 | 235418 | A | 0.8237 | G | 0.1763 | 30 |
| VMBQ01000052.1 | 235418 | A | 0.8571 | G | 0.1429 | 60 |
| VMBQ01000056.1 | 223883 | T | 0.1599 | A | 0.8401 | 1 |
| VMBQ01000056.1 | 223883 | T | 0.2148 | A | 0.7852 | 30 |
| VMBQ01000056.1 | 223883 | T | 0.2500 | A | 0.7500 | 60 |
| VMBQ01000068.1 | 364649 | T | 0.7850 | A | 0.2150 | 1 |
| VMBQ01000068.1 | 364649 | T | 0.7517 | A | 0.2483 | 30 |
| VMBQ01000068.1 | 364649 | T | 0.7083 | A | 0.2917 | 60 |
| VMBQ01000068.1 | 364654 | A | 0.7850 | G | 0.2150 | 1 |
| VMBQ01000068.1 | 364654 | A | 0.7517 | G | 0.2483 | 30 |
| VMBQ01000068.1 | 364654 | A | 0.7083 | G | 0.2917 | 60 |
| VMBQ01000079.1 | 23013 | T | 0.4642 | A | 0.5358 | 1 |
| VMBQ01000079.1 | 23013 | T | 0.4388 | A | 0.5612 | 30 |
| VMBQ01000079.1 | 23013 | T | 0.3529 | A | 0.6471 | 60 |
| VMBQ01000079.1 | 23034 | G | 0.4625 | A | 0.5375 | 1 |
| VMBQ01000079.1 | 23034 | G | 0.4456 | A | 0.5544 | 30 |
| VMBQ01000079.1 | 23034 | G | 0.3529 | A | 0.6471 | 60 |
| VMBQ01000093.1 | 16963 | A | 0.6623 | G | 0.3377 | 1 |
| VMBQ01000093.1 | 16963 | A | 0.6837 | G | 0.3163 | 30 |
| VMBQ01000093.1 | 16963 | A | 0.5511 | G | 0.4489 | 60 |
| VMBQ01000093.1 | 17031 | G | 0.6623 | T | 0.3377 | 1 |
| VMBQ01000093.1 | 17031 | G | 0.6862 | T | 0.3138 | 30 |
| VMBQ01000093.1 | 17031 | G | 0.5455 | T | 0.4545 | 60 |
| VMBQ01000093.1 | 17036 | G | 0.6656 | T | 0.3344 | 1 |
| VMBQ01000093.1 | 17036 | G | 0.6862 | T | 0.3138 | 30 |
| VMBQ01000093.1 | 17036 | G | 0.5511 | T | 0.4489 | 60 |
| VMBQ01000093.1 | 17235 | T | 0.6639 | G | 0.3361 | 1 |
| VMBQ01000093.1 | 17235 | T | 0.6871 | G | 0.3129 | 30 |
| VMBQ01000093.1 | 17235 | T | 0.5568 | G | 0.4432 | 60 |
| VMBQ01000099.1 | 631850 | G | 0.5940 | A | 0.4060 | 1 |
| VMBQ01000099.1 | 631850 | G | 0.6552 | A | 0.3448 | 30 |
| VMBQ01000099.1 | 631850 | G | 0.6867 | A | 0.3133 | 60 |
| VMBQ01000099.1 | 631873 | T | 0.5990 | A | 0.4010 | 1 |
| VMBQ01000099.1 | 631873 | T | 0.6586 | A | 0.3414 | 30 |
| VMBQ01000099.1 | 631873 | T | 0.6867 | A | 0.3133 | 60 |
| VMBQ01000099.1 | 631892 | C | 0.6003 | G | 0.3997 | 1 |
| VMBQ01000099.1 | 631892 | C | 0.6621 | G | 0.3379 | 30 |
| VMBQ01000099.1 | 631892 | C | 0.6905 | G | 0.3095 | 60 |
| VMBQ01000104.1 | 37842 | A | 0.6424 | T | 0.3576 | 1 |
| VMBQ01000104.1 | 37842 | A | 0.7376 | T | 0.2624 | 30 |
| VMBQ01000104.1 | 37842 | A | 0.7469 | T | 0.2531 | 60 |
| VMBQ01000117.1 | 407279 | C | 0.6839 | T | 0.3161 | 1 |
| VMBQ01000117.1 | 407279 | C | 0.7517 | T | 0.2483 | 30 |
| VMBQ01000117.1 | 407279 | C | 0.7557 | T | 0.2443 | 60 |
| VMBQ01000125.1 | 117433 | T | 0.8906 | C | 0.1094 | 1 |
| VMBQ01000125.1 | 117433 | T | 0.8511 | C | 0.1489 | 30 |
| VMBQ01000125.1 | 117433 | T | 0.7955 | C | 0.2045 | 60 |
| VMBQ01000125.1 | 117515 | G | 0.9141 | T | 0.0859 | 1 |
| VMBQ01000125.1 | 117515 | G | 0.8972 | T | 0.1028 | 30 |
| VMBQ01000125.1 | 117515 | G | 0.8125 | T | 0.1875 | 60 |
| VMBQ01000125.1 | 117531 | A | 0.9125 | T | 0.0875 | 1 |
| VMBQ01000125.1 | 117531 | A | 0.8936 | T | 0.1064 | 30 |
| VMBQ01000125.1 | 117531 | A | 0.8125 | T | 0.1875 | 60 |
| VMBQ01000136.1 | 382060 | C | 0.6969 | T | 0.3031 | 1 |
| VMBQ01000136.1 | 382060 | C | 0.6538 | T | 0.3462 | 30 |
| VMBQ01000136.1 | 382060 | C | 0.5926 | T | 0.4074 | 60 |
| VMBQ01000136.1 | 389224 | T | 0.8112 | A | 0.1888 | 1 |
| VMBQ01000136.1 | 389224 | T | 0.7276 | A | 0.2724 | 30 |
| VMBQ01000136.1 | 389224 | T | 0.6761 | A | 0.3239 | 60 |
| VMBQ01000136.1 | 389253 | G | 0.8112 | A | 0.1888 | 1 |
| VMBQ01000136.1 | 389253 | G | 0.7276 | A | 0.2724 | 30 |
| VMBQ01000136.1 | 389253 | G | 0.6705 | A | 0.3295 | 60 |
| VMBQ01000177.1 | 433496 | C | 0.7979 | T | 0.2021 | 1 |
| VMBQ01000177.1 | 433496 | C | 0.7645 | T | 0.2355 | 30 |
| VMBQ01000177.1 | 433496 | C | 0.7126 | T | 0.2874 | 60 |
| VMBQ01000179.1 | 47286 | C | 0.8123 | T | 0.1877 | 1 |
| VMBQ01000179.1 | 47286 | C | 0.7993 | T | 0.2007 | 30 |
| VMBQ01000179.1 | 47286 | C | 0.7035 | T | 0.2965 | 60 |
| VMBQ01000179.1 | 47405 | G | 0.8142 | T | 0.1858 | 1 |
| VMBQ01000179.1 | 47405 | G | 0.7972 | T | 0.2028 | 30 |
| VMBQ01000179.1 | 47405 | G | 0.7126 | T | 0.2874 | 60 |
| VMBQ01000188.1 | 223857 | T | 0.3447 | G | 0.6553 | 1 |
| VMBQ01000188.1 | 223857 | T | 0.4126 | G | 0.5874 | 30 |
| VMBQ01000188.1 | 223857 | T | 0.4244 | G | 0.5756 | 60 |
| VMBQ01000188.1 | 223874 | T | 0.4130 | A | 0.5870 | 1 |
| VMBQ01000188.1 | 223874 | T | 0.4685 | A | 0.5315 | 30 |
| VMBQ01000188.1 | 223874 | T | 0.5000 | A | 0.5000 | 60 |
| VMBQ01000192.1 | 196828 | T | 0.8177 | G | 0.1823 | 1 |
| VMBQ01000192.1 | 196828 | T | 0.7905 | G | 0.2095 | 30 |
| VMBQ01000192.1 | 196828 | T | 0.7414 | G | 0.2586 | 60 |
| VMBQ01000194.1 | 377087 | G | 0.7097 | A | 0.2903 | 1 |
| VMBQ01000194.1 | 377087 | G | 0.7397 | A | 0.2603 | 30 |
| VMBQ01000194.1 | 377087 | G | 0.7824 | A | 0.2176 | 60 |
| VMBQ01000205.1 | 114879 | A | 0.4766 | G | 0.5234 | 1 |
| VMBQ01000205.1 | 114879 | A | 0.5448 | G | 0.4552 | 30 |
| VMBQ01000205.1 | 114879 | A | 0.5517 | G | 0.4483 | 60 |
| VMBQ01000222.1 | 243753 | C | 0.4317 | A | 0.5683 | 1 |
| VMBQ01000222.1 | 243753 | C | 0.4586 | A | 0.5414 | 30 |
| VMBQ01000222.1 | 243753 | C | 0.5116 | A | 0.4884 | 60 |
| VMBQ01000229.1 | 197373 | A | 0.6139 | C | 0.3861 | 1 |
| VMBQ01000229.1 | 197373 | A | 0.6242 | C | 0.3758 | 30 |
| VMBQ01000229.1 | 197373 | A | 0.7303 | C | 0.2697 | 60 |
| VMBQ01000229.1 | 197399 | A | 0.6106 | C | 0.3894 | 1 |
| VMBQ01000229.1 | 197399 | A | 0.6208 | C | 0.3792 | 30 |
| VMBQ01000229.1 | 197399 | A | 0.7247 | C | 0.2753 | 60 |
| VMBQ01000229.1 | 197422 | A | 0.6914 | C | 0.3086 | 1 |
| VMBQ01000229.1 | 197422 | A | 0.7047 | C | 0.2953 | 30 |
| VMBQ01000229.1 | 197422 | A | 0.7921 | C | 0.2079 | 60 |
| VMBQ01000229.1 | 197482 | A | 0.6914 | C | 0.3086 | 1 |
| VMBQ01000229.1 | 197482 | A | 0.7047 | C | 0.2953 | 30 |
| VMBQ01000229.1 | 197482 | A | 0.7921 | C | 0.2079 | 60 |
| VMBQ01000229.1 | 197622 | G | 0.6122 | T | 0.3878 | 1 |
| VMBQ01000229.1 | 197622 | G | 0.6208 | T | 0.3792 | 30 |
| VMBQ01000229.1 | 197622 | G | 0.7191 | T | 0.2809 | 60 |
| VMBQ01000246.1 | 216692 | A | 0.8870 | G | 0.1130 | 1 |
| VMBQ01000246.1 | 216692 | A | 0.8297 | G | 0.1703 | 30 |
| VMBQ01000246.1 | 216692 | A | 0.8046 | G | 0.1954 | 60 |
| VMBQ01000253.1 | 258740 | G | 0.5339 | A | 0.4661 | 1 |
| VMBQ01000253.1 | 258740 | G | 0.5664 | A | 0.4336 | 30 |
| VMBQ01000253.1 | 258740 | G | 0.6236 | A | 0.3764 | 60 |
| VMBQ01000253.1 | 258788 | G | 0.5238 | A | 0.4762 | 1 |
| VMBQ01000253.1 | 258788 | G | 0.5638 | A | 0.4362 | 30 |
| VMBQ01000253.1 | 258788 | G | 0.6180 | A | 0.3820 | 60 |
| VMBQ01000256.1 | 193498 | A | 0.3194 | C | 0.6806 | 1 |
| VMBQ01000256.1 | 193498 | A | 0.2778 | C | 0.7222 | 30 |
| VMBQ01000256.1 | 193498 | A | 0.1707 | C | 0.8293 | 60 |
| VMBQ01000256.1 | 193512 | A | 0.3038 | T | 0.6962 | 1 |
| VMBQ01000256.1 | 193512 | A | 0.2708 | T | 0.7292 | 30 |
| VMBQ01000256.1 | 193512 | A | 0.1585 | T | 0.8415 | 60 |
